# Botanicals Against *Tetranychus urticae* Koch Under Laboratory Conditions: A Survey of Alternatives for Controlling Pest Mites

**DOI:** 10.3390/plants8080272

**Published:** 2019-08-07

**Authors:** Ricardo A. Rincón, Daniel Rodríguez, Ericsson Coy-Barrera

**Affiliations:** 1 Biological Control Laboratory, Universidad Militar Nueva Granada, Cajicá 250247, Colombia; 2 Bioorganic Chemistry Laboratory, Universidad Militar Nueva Granada, Cajicá 250247, Colombia

**Keywords:** *Tetranychus urticae*, resistance, botanical pesticides, acaricide, integrated pest management

## Abstract

*Tetranychus urticae* Koch is a phytophagous mite capable of altering the physiological processes of plants, causing damages estimated at USD$ 4500 per hectare, corresponding to approximately 30% of the total cost of pesticides used in some important crops. Several tools are used in the management of this pest, with chemical control being the most frequently exploited. Nevertheless, the use of chemically synthesized acaricides brings a number of disadvantages, such as the development of resistance by the pest, hormolygosis, incompatibility with natural predators, phytotoxicity, environmental pollution, and risks to human health. In that sense, the continuous search for botanical pesticides arises as a complementary alternative in the control of *T. urticae* Koch. Although a lot of information is unknown about its mechanisms of action and composition, there are multiple experiments in lab conditions that have been performed to determine the toxic effects of botanicals on this mite. Among the most studied botanical families for this purpose are plants from the Lamiaceae, the Asteraceae, the Myrtaceae, and the Apiaceae taxons. These are particularly abundant and exhibit several results at different levels; therefore, many of them can be considered as promising elements to be included into integrated pest management for controlling *T. urticae*.

## 1. Introduction

One of the most important pests in commercial crops worldwide is the polyphagous, two-spotted spider mite, *Tetranychus urticae* Koch. This mite is able to alter the physiological processes of plants, reducing the area of photosynthetic activity and causing the abscission of leaves in severe infestations [1]. The cost of damages caused by this pest in crops such as beans, citrus, cotton, avocado, apples, pears, plums, and many other horticultural and ornamental crops are estimated at over USD$ 4500 per hectare. Such costs correspond to 30% of the total cost of pesticides in crops of ornamental flowers. This constitutes a spending of almost 62% of the global market value on *T. urticae* Koch control based on data of 2008 [2]. The main tools used to control this pest are chemically synthesized acaricides. However, this mite is known to generate a resistance to these chemicals in a short period of time [3]. In addition, when the *T. urticae* Koch is exposed to sublethal pesticide levels, this mite has the ability to increase its reproduction rate, thus its populations increase in a shorter time [4]. Furthermore, many of the active ingredients in pesticide formulations are incompatible with the *T. urticae* Koch’s natural predators; consequently, when they are applied to crops, they suppress populations of predators that can contribute to the decrease of phytophagous mites [5].

Courtesy of the above-mentioned issues—together with problems related to environmental contamination, the risk for human and animal health, and phytotoxicity—it is necessary to complement the control of *T. urticae* Koch with tools other than chemically-synthesized acaricides, such as biological control and the use of botanical pesticides (plant extracts), a growing alternative for the control of this pest. From the perspective of locating new options for the control of two-spotted spider mites, the use of botanical pesticides represents a useful tool with minimal detrimental effects on the environment, a low residuality, a slight induction of resistance due to its complex matrix, and with fewer harmful effects on human health when compared to those of the chemically-synthesized acaricides. Therefore, in the present review, a survey is presented based on some characteristics of *T. urticae* Koch behavior in the presence of toxic substances. In addition, this review builds upon other studies in order to determine the biological activity of some botanical pesticides on the phytophagous mite *T. urticae* Koch under laboratory conditions.

## 2. Characteristics of *T. urticae*

The *T. urticae* Koch is the most abundant and the most widely distributed species of the genus *Tetranychus*. This genus presents a confusing taxonomy due to partial reproductive incompatibilities that have been found in some populations. It is known that, in certain cases, these incompatibilities are caused by species of bacteria from the genus *Wolbachia* [6].

The individuals of the *T. urticae* Koch are characterized by having two spots on their back (dorsal idiosome), green or brown coloration, and white or yellow colored legs [4]. They present sexual dimorphism, as males are smaller than females [4]. An important feature of this species is that it is is able to form a web on the plants in which it grows [4]. These mites feed initially on the leaves of the lower part of the plants, but they can later colonize the rest of them as the population grows. The damage they cause is observed in the form of chlorotic spots and, in some cases, the tanning of leaves and defoliation [4].

### 2.1. The Biology of T. urticae Koch

The life cycle of the family Tetranychidae includes the stages of egg, larva, protonymph, deutonymph, and adult [7], between each of which a quiescent state usually occurs. Their eggs are round, white, or translucent, and the duration of their cycle depends on the temperature, the relative humidity, and the host plant in which they develop. Under temperature conditions between 25 and 30 °C, the *T. urticae* Koch can complete its cycle between three and five days [8,9]. The eggs are approximately 0.13 mm in diameter. The larvae are spherical or oval in shape, generally greenish yellow with three pairs of legs, and their size is approximately 0.16 mm in length. The protonymphs have an oval shape and a pale green color. They are distinguished from larvae by having four pairs of legs, and their length is approximately 0.2 mm. In the case of deutonymphs, they reach a length of approximately 0.3 mm and have a yellow or light brown color. At this stage, two dark brown spots usually appear on the dorsal level. On the other hand, adults have a globular or oval shape and range from pale green to reddish yellow in color; adults present two red or dark brown spots on the idiosome. Males are smaller than females, with lengths of 0.4 and 0.5 mm, respectively [7,10]. This species is arrhenotokous [8], which increases the probability that a female will mate with her offspring. According to some authors, its high genetic variability allows it to adapt quickly and decreases its probability of expressing deleterious mutations [9].

### 2.2. Characteristics of Resistance of T. urticae Koch to Acaricides

The *T. urticae* Koch is a widespread polyphagous pest that attacks more than 1100 different plant species [9,11], making it one of the main phytosanitary problems for many crops. This trait is owing (among other reasons) to its capacity for quickly generating resistance to synthetic acaricidal products [12]—from two to four years of new active ingredients [9]—even after a few applications of the active ingredient [11].

This resistance capacity to pesticides of the *T. urticae* Koch has encouraged some researchers to carry out several studies regarding their genetic characteristics in response to the pressure generated by the use of acaricides. Such is the case of Grbić et al. (2011) [9], who carried out a deep analysis of the *T. urticae* Koch genome. They found that more than 10% of their genome comprises transposable elements (9.09 Mb). In the same study, they also observed the presence of several families of genes involved in digestion, detoxification, and transport of xenobiotic compounds with a unique composition. Eighty-six genes encode for cytochromes P450, a group of 32 genes encode for glutathione S-transferases (GST) (12 of these are believed to be unique to vertebrates), and 39 genes encode for drug-resistant proteins of the ABC transporters type (ATP-binding cassette). This repertoire of transporter proteins greatly exceeds the number presented by crustaceans, insects, vertebrates, and nematodes.

All these detoxifying enzymes are closely related to the resistance of *T. urticae* Koch, but this is not the only mechanism used by these mites to counteract the effect of xenobiotics. A set of mutations in the action points of pesticides is another way they are able to mitigate the effect of these compounds. Demaeght et al. (2014) [13] reported a resistance case for this species when there was a mutation in quitin synthase 1, which is the target enzyme of etoxazole. Additionally, because of its similarity to the mechanism of action of hexythiazox and clofentezine, this mutation can cause a cross-resistance to these products. Table 1 shows an example of the effects of 10 different acaricides on four different populations of *T. urticae* Koch in the state of Pernambuco (Brazil) [14]. This information demonstrates the ability of this pest to counteract the effects of different active ingredients, showing variable responses to the same compounds in different regions.

#### Function of Detoxifying Enzymes

Cytochrome P450 has been extensively investigated, as it is the most important group of detoxifying proteins in arthropods [15]. This enzyme group has been linked to cases of resistance in the common fly, *Musca domestica* L., with resistance to those furanocoumarins produced by a host plant in *Papilio polyxenes* Fabricius [15], and in cases of resistance to abamectins in *T. urticae* Koch [16]. One of the characteristics of this group of proteins in arthropods is their inducibility over time, which is proportional to the consumption of certain toxic compounds from the plants that serve them as food. Such is the case of the *Spodoptera frugiperda* Smith. In this species, it was demonstrated that, when consuming a diet containing indole-3-carbinol, in time and with the increase in the concentration of this compound, there was an increase in the production of P450 enzymes [15].

Another group of proteins that is important in the response to xenobiotics is the Glutathione-S-Transferases (GST) family. Among these proteins, two enzymes belonging to the delta class—tuGSTd10 and tuGSTd14—and one of the mu class—tuGSTm09—are present in *T. urticae* Koch. They are strongly associated with mite resistance to the active ingredient abamectin [17]. Similarly, Pavlidi et al. (2017) [18], through molecular docking analysis and implementation of HPLC-MS, deduced that the active ingredient cyflumetofen and its de-esterified metabolite could be transformed by the enzyme TuGSTd05 in the same mite species.

On the other hand, Merzendorfer (2014) [19] and Dermauw and Van Leeuwen (2014) [20] mentioned the presence of 104 genes belonging to subfamilies of ABC genes in *T. urticae* Koch. This number is higher than that of other different species such as *Homo sapiens* L., *Apis mellifera* L., *Drosophila melanogaster* Meigen, *Anopheles gambiae* Giles, *Bombyx mori* L., *Tribolium castaneum* Herbst, *Pediculus humanus* L., *Daphnia pulex* Leydig, *Caenorhabditis elegans* Maupas, and *Saccharomyces cerevisiae* Meyen ex EC Hansen, demonstrating its importance within this species. This group of genes has also been related to the development of elytra and wings in some insects and to the transport of certain drugs of hydrophobic origin. The type of transport of compounds of these proteins has been elucidated through models constructed by crystallography, for which it is known that they act as proteins of import, export, or as flipases [19].

### 2.3. Relationship Between Resistance and the Host Plant

The different mechanisms of resistance presented by the *T. urticae* Koch suggest that these adaptations may not be due exclusively to the pressure generated from the use of pesticides. This question was asked by Dermauw et al. (2012) [21], who made an interesting finding when studying the transcriptome of resistant and susceptible strains of the *T. urticae* Koch in the presence of different host plants.

**Figure 1:**
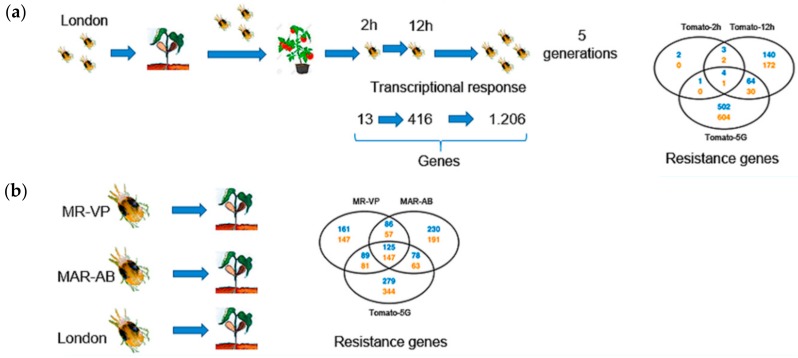
A graphical representation of the study performed by Dermauw et al. (2012) [21]. (**a**) represents the transcriptional changes in the susceptible London strain of the *T. urticae* Koch when changing host plant. (**b**) represents the number of genes expressed in two resistant strains and the susceptible London strain of *T. urticae* Koch after 5 generations from the relocation to another host plant. The scheme was constructed by R.A. Rincón for this review from the data published by Dermauw et al. (2012) [21].

In that study, they demonstrated that a susceptible strain of this phytophagous mite was capable of expressing diverse deactivated genes when it was relocated from a bean to a tomato as its host plant [21]. In addition, the number of expressed genes that are related to the generation of resistance increased considerably, going from 13 genes—expressed after two hours from host plant change—to 1206 genes after five generations. On the other hand, they compared the transcriptome of two resistant strains and that of the susceptible strain developed in the tomato. They also found that both mite strains shared the expression of a significant number of genes related to resistance (Figure 1). This seems to indicate that there is a strong relationship between the resistance mechanisms developed by the *T. urticae* Koch and its host plants. These mechanisms may be similar to those developed by this species to face exposure to different pesticides.

Evidence of the resistance capacity of this phytophagous mite is shown in Table 2. A list of important pest arthropod species is shown, reporting the number of active ingredients to which they developed resistance until the year 2012 [22,23]. The list is led by the *T. urticae* Koch, a species that showed a reported resistance to 93 active ingredients until that moment.

Owing to the large number of reports of resistance existing for the *T. urticae* Koch, some studies have provided important information and promising aspects in terms of understanding the resistance with promising results. Such is the case of the research conducted by Demaeght et al. (2013) [24] concerning cross-resistance. They studied two *T. urticae* Koch strains that were resistant to Spirodiclofen—an active ingredient belonging to group 23 of the IRAC (i.e., inhibitors of acetyl CoA carboxylase). Although strains appeared to be strongly resistant to this ingredient, they had a very low cross-resistance to spirotetramat and spirodiclofenenol. This information could serve as a base for the understanding of some routes of resistance-generation in this phytophagous mite, because they demonstrated that the spirodiclofen detoxification route affects—at least partially—all of the tetranic and the tetronic acid derivatives in the *T. urticae* Koch.

In the same study, Demaeght et al. (2013) discarded resistance to spirodiclofen by active site mutations after aligning the sequences of active sites from target proteins with BlastP [24]. However, when microarrays were made to express the genome of the studied strains and subsequently compared, they found similarities in several genes expressed among the spirodiclofen resistant strains, which were identified as P450 family proteins, carboxylesterases, glutathione S-transferases, transport proteins, lipocalins, and several proteins without homology in the available databases. This fact demonstrated that this detoxifying route is strongly related to the response of the *T. urticae* Koch to this ingredient.

On the other hand, Kwon et al. (2012) [25] detected a fitness decrease of *T. urticae* Koch strains that demonstrated Monocrotophos resistance. Although the presence of more than one mutation increased the resistance up to 1165-fold, these modifications in genes significantly decreased the catalytic capacity of acetyl cholinesterase, thus gene overexpression seems to be necessary in order to compensate for deficiency acquired by resistance-conferring mutations to the acaricide.

## 3. Control Strategies for *T. urticae* Koch

In agricultural crops, the main pest control method used is the spraying of solutions based on chemically synthetic products such as insecticides and acaricides [26]. Although this method has been effective in some cases for *T. urticae* Koch control, it has also demonstrated serious limitations and disadvantages, especially due to *T. urticae* Koch’s high reproductive potential. This peculiarity encourages farmers to use acaricides in larger volumes and doses, causing high levels of toxic waste in fruits, the development of resistant populations, the intoxication of mammals, and the destruction of beneficial organisms [23,27,28].

Another strategy used for *T. urticae* Koch management is biological control. Among the predators of this pest are some mites of the family Phytoseiidae. Within this family, two predators stand out—the *Neoseiulus californicus* McGregor and the *Phytoseiulus persimilis* Athias-Henriot. These mites are characterized by consuming a large number of prey at adequate conditions and having high reproductive rates and a capacity for rapid development [4]. Other natural predators that are less commonly used for the control of this mite are the beetle *Stethorus punctillum* Weise (Coccinellidae) and the *Conwentzia psociformis* Curtis (Neuroptera: Coniopterygidae)—which are found naturally in Spain [29]—purely to mention some of the predators of this phytophagous species. Additionally, the fungus *Neozygites floridana* Weiser and Muma has also exhibited significant control over the *T. urticae* Koch, but some difficulties in cultivation have hampered its use [30]. However, other fungi such as the *Lecanicillium lecanii* Zimmermann and the *Beauveria bassiana* Bals.-Criv., as well as the bacterium *Bacillus thuringiensis* Berliner, have been commercially used for the management of the two-spotted spider mite with positive effects.

### 3.1. Other Methods for T. urticae Koch Control

As complementary strategies, these mites are controlled in some crops through the application of water washings and the manual massaging of the affected leaves using water and soap in order to remove the mites from the plant, kill them mechanically, and break their webs. Within the strategies used for controlling the *T. urticae* Koch, biopesticides based on plant extracts or phytochemicals are considered to be another alternative to chemically-synthesized acaricides [31,32], which have also emerged as a complement to traditional management. This has allowed the development of commercial products with formulations based on substances of natural origin, such as CinnAcar^®^, Biodie^®^ and PHC Neem^®^, which are produced from compounds and mixtures isolated from plant extracts. As an example for this case, they have demonstrated compatibility with the natural predator *Tamarixia radiata* Waterston (Hymenoptera: Eulophidae)—parasitoid of the *Diaphorina citri* Kuwayama (Hemiptera: Psyllidae)—thus these formulations may constitute excellent alternatives to be included into integrated management programs (IPM) of the so-called “Asian citrus psyllid” [33]. Therefore, the fact that these botanical pesticides are compatible with natural predators becomes an advantage in the control of pests and constitutes an additional tool that can be used in integrated pest management strategies.

An essential prerequisite for success when using extracts as a control strategy for pests is their compatibility with other management strategies. Within the context of the IPM, a relevant issue is the evaluation of how this type of product can affect biological control agents. In the particular case of the *T. urticae* Koch, a question arises about how phytoseiid mites that have been successfully used as a control strategy could be affected—a topic that has been explored by different researchers. Among the botanical pesticides, probably the most used are the neem derivatives, a trend that is also present in the case of the *T. urticae* Koch. A moderate reduction in female survival and fecundity in response to Azadirachtin use on *P. persimils* Athias-Henriot was reported by Duso et al. (2008) [34], although a positive shift in favor of the predator in terms of the predator–prey interaction can be inferred, since azadirachtin was more toxic to the *T. urticae* Koch. A moderate effect was also reported by Spollen and Isman (1996) [35], who found a maximum mortality of 14% in *P. persimils* Athias-Henriot adults sprayed with neem extract. Although variables such as egg eclosion, the mean number of eggs laid per female, and differences in preference between treated or untreated leaves were not found, the authors concluded that neem-derived insecticides could be effective and safe. Neem pesticides have exhibited few negative impacts on the phitoseids *N. californicus* McGregor [36], *Euseius alatus* De Leon [37], and *Phytoseiulus macropilis* Banks [36,37]. On the other hand, the negative effects of NeemAzal-T/S in terms of its potential impact on populations of the predator *Metaseiulus occidentalis* Nesbitt were reported by Yanar (2019) [38], who recommended the use of low concentrations of this product in cases where the *M. occidentalis* is a relevant component of IPM. Regarding another plant species used to obtain botanicals, crude extracts of *Artemisia judaica* L. exhibited acaricidal bioactivity against *T. urticae* Koch in terms of LC_50_, while its negative impacts upon *P. persimils* Athias-Henriot were clearly lower, suggesting that such extracts are compatible with the predaceous mite [39]. Similarly, a promising toxic effect of the *Melissa officinalis* L. on *T. urticae* Koch has been reported, along with an LC_50_ for the *N. californicus* McGregor, which is comparatively extremely lower. Undertaking compatibility evaluations between extracts and natural predators is essential, since there is no reason to generalize slight or innocuous effects of these products on said beneficial organisms. Commercial formulations and application rates similar to those used by farmers are needed in order to obtain results with more predictive value in respect of those expected in the field. Sublethal effects will also be a subject of relevant research in the future, because, although many of the evaluations that demonstrate little or no effect on the natural predators have been carried out in adults, the sublethal effects could raise compatibility issues that are not evident when restricting evaluations to adult individuals [40].

### 3.2. The Use of Plant Extracts for the Control of T. urticae Koch in The Field

There are many studies regarding the use of plant extracts for the control of the *T. urticae* Koch. Although many of these trials have delivered successful results, others have not demonstrated the level of expected control over this mite species. For that reason, a greater understanding of the mechanisms of action presented by molecules that demonstrate biological activity on these mites and the way in which these molecules interact is highly required. In addition, the toxic effects of such molecules are generated in many cases by the presence of several toxic compounds contained in the same extract, which act in a synergistic manner. An understanding of these factors will help to foster a broader understanding of the use of this tool in the control of the two-spotted spider mite. Further studies must take into account the results of the studies developed thus far, which have delivered promising results, not just in terms of the toxic effects demonstrated on these mites, but also in terms of sublethal effects such as low fecundity and repellency.

#### 3.2.1. Methods for the Evaluation of Extracts under Laboratory Conditions

The methods of testing the effects of extracts are very much the same as those used thus far for evaluations of chemical compounds. An important prerequisite for making appropriate evaluations is having a susceptible population of individuals. Generally, this population can be obtained by rearing individuals that have not been exposed to any type of chemical substance with a possible acaricidal effect for a considerable number of generations [5,41,42,43]. Additionally, the origin and the type of the selected plant material must be clearly defined in order to ensure the repeatability of results. Hence, correct taxonomic classification, location, season of the year, time of day, phenological stage, and organs to be collected and processed in order to obtain the extracts affect the particular composition of the tested botanical and influence the acaricidal activity [5,44,45,46]. Finally, the type of preparation and the extracting protocol are also crucial steps for obtaining a standardized mixture of plant-based compounds, which would be the source of effective botanical-based acaricidal or repellent agents. Thus, solid–liquid (S–L) extractions—i.e., the selected plant material directly enters into contact with the extracting solvent during a defined period through a continuous (maceration) or discontinuous (percolation or Soxhlet) procedure—are the most commonly used method for obtaining different types of extracts, depending on the polarity of the extracting solvent. In order of polarity, water, water/ethanol mixture (hydroalcoholic), ethanol/methanol, chloroform, ethyl acetate, and hexane are the most commonly used solvents for extractions. Other types of preparations are essential oil (usually obtained by steam distillation) or low-polar/volatile extracts (afforded by hydrodistillation, supercritical fluid extraction, microwave or ultrasound-assisted hydrodistillation, among others) [47]. The physicochemical nature of these naturally occurring compounds, which are present in the preparation (extract or essential oil), is the critical prerequisite information required to identify the extracting procedure. The purification or the isolation of the active principles requires several steps, usually using preparative techniques such as column chromatography under a bioguided fractionation strategy—although the isolated compounds might be separately assessed after a conventional purification protocol. In any case, these efforts could affect upon acaricidal rather than repellent activities to facilitate mite control, but this choice depends of the aims of use. Essential oils often exhibit repellent activity in comparison to extracts, owing to their volatile nature.

#### 3.2.2. Bioassays

The purpose of bioassays is to determine the effect of a given agent on the physiology of an organism, which, in the context of acari research, is generally associated with determining the toxicity of a chemical compound—or resistance to it—either in the field or in laboratory conditions [48]. Repeatability of results, practical facilities, and conditions as similar as possible to those under which the acaricide will be used are desirable [49]. In the case of mites, a small size and fast movement are aspects conditioning the bioassay design. The main aspects of some common bioassays used for the evaluation of botanicals on *T. urticae* Koch adults (generally females) and their advantages and disadvantages are described below.

##### Slide Dip Methods

An initial method was described by Voss (1961) [50] as part of an acaricide screening procedure. Double-sided Scotch^®^ tape is adhered on one of its sides to a microscope slide. It is important to avoid bubbles or empty spaces between the tape and the glass, because deposits of the substance under evaluation could be formed, which could affect the test results [51]. After this, the mites must be affixed to the other side of the tape by the dorsal part of the hysterosoma. A fine brush is usually used to transfer individuals to the tape. The slides are then dipped into the solution containing the toxicant for 5 s [48,51] and, after this, are placed on a paper towel. It is important to remove any excess of liquid with filter paper. After this, the slides are placed on trays covered with slightly moistened disposable towels, which must then be taken into controlled conditions. The mortality criterion in the different methods is usually an absence of movement when the individual is gently prodded with a fine brush. High control mortalities due to desiccation and an absence of food are common in this method for times of evaluation greater than 24 h, limiting the accuracy of the response parameters. Furthermore, individuals are exposed to toxicants in an artificial substrate, and in some cases, problems distinguishing alive and dead mites arise [48]. Despite such problems, this method has been used repeatedly to determine the effect of botanicals on adults of the *T. urticae* Koch, in some cases considering evaluation times of 24 h [52], but in other cases employing higher evaluation times [41,52,53,54,55,56,57]. An advantage of this method is that the results obtained are highly reproducible. It can also be modified by employing a spray tower to supply the toxicant, which allows for a better coverage [58].

##### Petri Dish Methods

The main variant is the Petri Dish Residue-Potter Tower Method (PDR-PT), in which the bottom and the top inner surfaces of a Petri dish are sprayed with the toxicant using a Potter Tower and allowed to dry for around 30 min at room temperature. After this, the individuals are transferred to the dishes using a fine brush. For this method, high mortalities after 48 h have been reported, thus it is advisable to restrict the evaluation time to shorter periods, such as 24 h [59]. The petri dish methods have been used in some cases for the evaluation of botanicals against *T. urticae* Koch [3,60].

##### Leaf Disc Methods

For this kind of bioassay, leaf discs of variable diameter (approximately 20 mm) are cut from leaves of several plant species, such as beans [1,43,46], peaches [48], or roses [3], and placed upside down in a Petri dish containing moistened cotton wool when bean or rose leaf discs are used or a semi-solid agar pad in the case of peach leaf discs. A variable number of adults (between five and 20) must be transferred to each leaf disc using a fine brush. The experimental assembly is maintained without disturbance for at least one hour before the spraying of the toxicant is performed. This spraying can be performed using an airbrush, provided the required distance—as well as the number of drops/cm^2^ and the pressure—may be adequately standardized [61], which corresponds to the basic Leaf Disc Direct Method (LDD). This method can be improved employing the Potter spray tower, derivating in the so-called “Leaf Disc Direct-Potter Tower Method” (LDD-PT) [48]. This device was developed by C. Potter from the Rothamsted Experimental Station [62], and it is recognized as a reference standard for making chemical sprays under laboratory conditions, since it enables the achievement of an even deposition of spray in the target area. The LDD and the LDD-PT methods can also be used to evaluate the effect of a residual film of the toxicant on adults placed on a sprayed surface (such as a leaf disc in this instance). In this case, the procedures are named “Leaf Disc Residue Method” (LDR) and “Leaf Disc Residue-Potter Tower Method” (DR-PTM) [48]. Both a direct spray and the residual film are intended to evaluate the toxic effects generated by contact between the individual mites and the test substance. After the spray, Petri dishes are kept uncovered for around 30 min, which allows for the drying of the leaf disc surface. They are then covered and placed under controlled conditions. Generally, mites that cannot walk a distance equivalent to their body length are considered dead. Since the leaf disc method implies the presence of the natural substrate of spider mites, it can be considered to have a greater similarity with field conditions than the slide dip or the petri dish methods. However, one drawback is the escape of individuals. This problem intensifies when the toxicant requires a prolonged time to act or when it should be ingested in the feeding process. The fate of individuals that escape is uncertain, thus the most advisable procedure is to discard them in the analysis; to consider them as part of the mortality rate would not be justifiable [48]. An alternative approach is the development of methods that do not allow for the escape of individuals, as proposed by Bostanian et al. [63]. In their setup, a large leaf disc (50 mm in diameter) is placed upside down and tightly fitted to the bottom of a plastic Petri dish of the same diameter, thus it occupies the whole dish. The base of each petri dish contains thinly moistened cotton wool (1.5 mm in thickness) to prevent desiccation. A circular window of 28 mm is cut in the top of the Petri dish to facilitate air circulation and avoid condensation. For bioassays involving tetranychyds, they recommend covering the window with a 40 μm polyester mesh screen to avoid run-off. The edges of the Petri dish bottoms are wrapped with masking tape to ensure a strong grip on the top, preventing the escape of individuals. A small hole in the lower half of the Petri dish allows the petiole to protrude outwards, where it must be covered with a wet cloth. This method enables observations for a period as long as nine days, which makes it suitable for slow- and fast-acting reduced-risk toxicants. A different variant of the leaf disc method is the Leaf Disc-Residue Dipping Method (LDR-D), in which the leaf discs are dipped into the solution containing the toxicant [64]. Although estimations of lethal concentrations obtained by this method are less precise when compared to the LDD-PT, this fact could be explained by an uneven distribution of residues on the leaf surface. The leaf disc methods have been widely used in several trials of botanicals against *T. urticae* Koch [5,40,54,55,56,57,58,59,60,61,62].

##### Leaf Absorption Method

In this method, the leaf is placed in some kind of recipient containing the toxicant solution in order to allow the absorption by the leaf for an adequate period (usually around 72 h). The leaf is then located in a Petri dish containing agaropectin to prevent desiccation, and the mites are transferred onto the leaf, where they are allowed to feed by 24 h, and mortality is then evaluated. The design of the experimental unit should consider alternatives to prevent the escape of mites, as discussed in the leaf disc method [42,63].

##### Whole Plant Direct Method

The purpose of this kind of bioassay is to evaluate the direct effect of toxicants under conditions as similar as possible to those of the field. Young bean plants with 2–3 leaves can be used, and the apical part of the plant must be removed to prevent the appearance of new leaves, which have not received the treatment. Adult females are then placed in the plants long enough before performing the application in order to allow oviposition. Alternatively, a specific number of immature stages and adults can be placed in the plants. The spray of the toxicant is made using an atomizer, considering an application volume similar to the required under crop conditions. The number of eggs, nymphs, and adults is recorded at predefined evaluation times, usually between 5 and 10 days [65,66]. This method has been also used to evaluate repellency [67].

##### Filter Paper Difussion Methods (Fumigant Bioassays)

This kind of assay is designed to evaluate the fumigant action of toxictants, thus it is essential to avoid any direct contact between individuals and the toxicant. The setup is similar to that employed in the leaf disc method, but the bottoms of the Petri dishes are covered using a tight-fitting lid with a fine wire sieve. The toxicant is applied to filter papers, which should be allowed to dry before being placed over the wire sieve [68]. In some cases, the paper is attached to the downside of the lid with a small quantity of solid glue that should not affect individuals [42].

### 3.3. Studies Carried Out for the Control of T. urticae Koch from Plant Extracts Grouped by Plant Families

The investigations carried out, which focused on the effects of biopesticides on *T. urticae* Koch, have led to the identification of a large number of plant extracts with acaricidal, repellent, and deterrent properties. Below are descriptions of some species—grouped by plant families—whose plant extracts have been used in laboratory studies that have exhibited their biological activity on the two-spotted spider mite (the information is summarized and complemented in Table A1 in the Appendix A).

#### 3.3.1. Family Amaranthaceae

This family has aroused interest in different areas such as traditional and alternative medicine, given the properties that have been identified in some of the species that comprise it. Such is the case of *Achyranthes aspera* L., whose secondary metabolites have antinociceptic activity [69], or *Chenopodium ambrosioides* Mosyakin et Clemants, which has toxic effects that have been studied in some human parasites [70].

Due to these toxic effects, Hiremath et al. (1995) [71] evaluated the acaricidal effect of the extract of this plant. They compared the activity of the methanolic extracts obtained from 21 different species of African plants against *T. urticae* Koch adults using the leaf immersion method. Among the most active extracts, the whole plant of *Celosia trigyna* Linn. exhibited the highest biological activity, causing mortality rates between 40% and 60% of evaluated mites.

Chiasson et al. (2004) [41] also evaluated the acaricidal effects of a species of this family. They studied the effect of an emulsifiable concentrate—obtained from *Chenopodium ambrosioides* Mosyakin et Clemants essential oil—on adults and eggs of the *T. urticae* Koch and the *Panonychus ulmi* Koch and compared it with the effect obtained from the use of commercially available products. The products were applied with an airbrush on females that were placed on microscope slides with glue. In the case of eggs, the application was made on the eggs previously laid by females on leaf discs located within Petri dishes. Thus, a dose of 0.5% produced a mortality of 94.7% in females, which was higher than that obtained from the Neem extract (22.1%). Otherwise, hatching was diminished on days five and nine after application. This hatching effect was lower in treatments with Neem, Abamectin, and insecticide soap. A lower effect was observed for an ethanolic extract from seeds of *Chenopodium quinoa* Willd. on adult females and nymphs of this mite, exhibiting an LD_50_ of 1.24% w/v [72].

Two years later, Shi et al. (2006) [52] evaluated the effect of *Kochia scoparia* (L.) Schrad extract on *T. urticae* Koch, *T. cinnabarinus* Boisdu-Val, and *T. viennensis* Zacher using three different solvents for extracting the compounds contained in the plant material: methanol, chloroform, and petroleum ether. The mortality trials were carried out using three different methodologies: (1) the slide dip method measuring mortality after 24 h of immersion, (2) the LDD-PT, and (3) the leaf absorption method. Using these methodologies, the highest mortality of the *T. urticae* Koch was obtained with the chloroform-soluble extract, which exhibited a 78.86% average mortality and an LC_50_ of 0.88 using the dipping method, in which mites were glued to an adhesive tape.

#### 3.3.2. Family Amaryllidaceae

This family is studied widely due to its potential uses in the control of human diseases [73] as well as its antitumor [74] and insecticides properties [75]. Abbassy et al. (1998) [76] determined the LC_50_ of the alkaloidal extract, the ethanolic extract, and the essential oil of the bulb of the ornamental plant *Pancratium maritimum* L. (Amaryllidaceae) on the *T. urticae* Koch, whose values were 0.2%, 0.36%, and 1.5%, respectively.

The insecticidal properties demonstrated by various studies led Attia et al. (2011) [77] to expose adult *T. urticae* Koch females to different concentrations of garlic extract (*Allium sativum* L.). These concentrations ranged between 0.46 and 14.4 mg/L using the Potter Tower application. After the bioassays, they determined the LD_50_ and the LD_90_, whose values were 7.49 and 13.5 mg/L, respectively. On the other hand, they concluded that fecundity was reduced by using the concentrations of 0.36 and 0.74 mg/L. Geng et al. (2014) [78] measured the toxicity by the contact and the repellency of the garlic extract at 20, 10, 5, 2.5, and 1.25 g/L. From these tests, they found that treatment with 20 g/L caused a 76.5% mortality rate on mites at 48 h after its application. Additionally, with the obtained data, they calculated the regression equation of toxicity as Y = 1.3 x + 3.9. They were also able to determine the LD_50_ value, which corresponded to 7.2 g/L. Furthermore, the repellencies were found to be 95.6% and 65.2% at extract concentrations of 10 g/L and 20 g/L, respectively.

#### 3.3.3. Family Annonaceae

Within this group of plants, the presence of several important secondary metabolites involved in the communication of arthropods and plants’ defenses against the attack of pests has been identified [79]. However, Ohsawa et al. (1991) [80] obtained negative results when using *Annona glabra* L. seed extract on *T. urticae* Koch eggs. During their experiment, they dissolved 10 mg of the extract in acetone (1 mL) and applied 0.5 mL of the solution to a bean leaf where the eggs were laid. After this, they noticed that the extract demonstrated no impact on mortality rates, deterrence in feeding, or mite growth.

Pontes et al. (2007) [44] also demonstrated the acaricidal activity of the essential oils of this family of plants, but in this case, they used the species *Xilopia serícea* A.St.-Hil., which was evaluated on *T. urticae* Koch. Using gas chromatography–mass spectrometry (GC–MS), they identified the compounds present in both leaves and fruits, finding mostly monoterpenes and sesquiterpenes. When comparing their acaricidal activity, they concluded that the essential oils of the leaves exhibited a greater toxicity than those obtained from the fruits.

#### 3.3.4. Family Apiaceae

Plants of this family are widely used within the diet of different human communities [81], although their nature is so varied that many species have been used as pesticides and repellents [82]. For example, Choi et al. (2004) [42] tested the essential oils of 53 plants to determine their acaricidal potential on *T. urticae* Koch eggs and adults. Among these oils, the highest toxicity was exhibited by species of the family Apiaceae—i.e., *Carum carvi* L.—since a 100% mortality rate of adult mites was obtained. To carry out this study, the researchers conducted bioassays by diffusion on filter paper, avoiding any direct contact between the oil and the mites. The tests were developed in a plastic container (4.5 × 9.5 cm) at a concentration of 14 × 10^−3^ μL/mL air.

Tsolakis and Ragusa (2007) [83] studied the effect of a mixture of essential oils from the *C. carvi* L. with potassium salts of fatty acids on the *T. urticae* Koch and one of its predators, *Phytoseiulus persimilis* Athias-Henriot. This combination proved to be very selective, since it generated a mortality rate of 83.4% in *T. urticae* Koch females compared to a 24% mortality rate in *P. persimilis* Athias-Henriot females. Besides, the product also caused a decrease in the intrinsic growth rate of the phytophagous mite while having no effect on the growth rate of the predator. Approximately four years later, this same essential oil was tested by Han et al. (2010) [68] on the same species of mite. In this case, by using mortality bioassays by vapor phase to evaluate fumigant effect (see section of Myrtaceae Family), they established an LD_50_ of 22.4 μg/cm^3^ air.

Among works carried out with plants of this family, Attia et al. (2011) [43] showed that the *Deverra scoparia* Coss. & Durieu essential oil has an acaricidal effect and decreases the fecundity of the *T. urticae* Koch. In the same study, they isolated the components of the oil and tested them individually on the pest, obtaining the highest toxicities with the compounds α-pinene, Δ^3^-carene, and terpinen-4-ol. Amizadeh et al. (2013) [84] also decided to evaluate the effect on two species of this family of the inhalation of essential oils. For this purpose, they carried out tests to determine the fumigant activity of *Heracleum persicum* Desf. Ex. Fisch. essential oils and *Foeniculum vulgare* Mill. seeds on adult females and eggs of the *T. urticae* Koch. The LD_50_s were 3.15 μL/L and 1.53 μL/L for females and eggs treated with *Heracleum persicum* Desf. Ex. Fisch. essential oil, respectively, and 5.75 μL/L and 1.17 μL/L for females and eggs treated with *Foeniculum vulgare* Mill. essential oil, respectively. Other essential oils obtained from Apiaceae plants having acaricidal activity on *T. urticae* Koch were *Cuminum cyminum* L. (seeds) and *Ferula gumosa* Boiss (leaves), showing LD_50_ values of 3.74 and 6.52 μL/L air, respectively [85,86].

On the other hand, Pavela (2015) [65] tested acaricidal and ovicidal effects of the methanolic extract of *Ammi visnaga* (L.) Lamarck seeds on *T. urticae* Koch. The efficacy in terms of adult mortality rates increased over time, with LD_50_s (after 72 h from the time of application) estimated at 17, 10, and 98 μg/cm^2^ for the extract and its two major compounds, khellin and visnagin (furanochromenes), respectively. Moreover, the extract and the two isolated furanochromenes inhibited the development of eggs and caused their mortality, with LD_50_s of 13.3, 0.5, and 1.8 μg/cm^2^ for the extract, the visnagin, and the khellin, respectively. The application of the extract to leaves infested with *T. urticae* Koch achieved a reduction of the number of individuals in all stages of development. The concentration of 10 mg/mL showed the highest efficacy, which was 98.5% on the tenth day since the application. The terpenes isofuranodiene and germacrone, isolated from *Smyrnium olusatrum* L. inflorescences, also exhibited toxicity on this mite (LD_50_s = 1.9 and 42.7 µg/mL, respectively) [87].

#### 3.3.5. Family Asteraceae

There have been numerous studies carried out with species from this group to evaluate their acaricidal activity. First, Chiasson et al. (2001) [45] evaluated the essential oils of two plant species known as potential pesticides—*Artemisia absinthium* L. and *Tanacetum vulgare* L.—to determine their acaricidal activity against the *T. urticae* Koch. The oils were obtained via a microwave-assisted process (MAP), distillation in water (DW), and by direct distillation with steam (DDS), and their relative toxicities were tested by direct contact. All oils were tested at 1%, 2%, 4%, and 8% as emulsions prepared in water with 9% denatured ethanol and 0.32% Alkamul EL-620 as emulsifier, and mite mortality was evaluated after 48 h.

The three oils of *A. absinthium* L. were toxic to the *T. urticae* Koch; however, there were differences in their levels of toxicity. For example, the oil extracted by MAP and DW methods caused 52.7% and 51.1% mortality in the mites, respectively, while the oil obtained by DDS produced a mortality rate of 83.2%. Consequently, the LC_50_ of the oil extracted by DDS was lower (0.043 mg/cm^2^) than those obtained by MAP (0.134 mg/cm^2^) and by DW (0.130 mg/cm^2^). The extracts of *T. vulgare* L. obtained by DW and DDS exhibited greater acaricidal activity than the extract prepared by the MAP method. At a concentration of 4%, oils delivered mortality rates of 60.4%, 75.6%, and 16.7%, respectively. The chemical analysis of the extracts of *T. vulgare* L. indicated that the compound *p*-thujone is the major compound in the oil (>87.6%) and probably contributes significantly to its acaricidal activity. Additionally, the acetone extract from leaves of *Artemisia judaica* L. exhibited an LD_50_ of 0.56 μg/mL against adult females [39].

Trials that have shown acaricidal activity within this family have also identified important compounds in essential oils that may play a role in the toxic activity against the *T. urticae* Koch. One of these cases was developed by Attia et al. (2012) [46], who identified the terpinen-4-ol compound in the *Santolina africana* Jord. & Fourr. essential oil. This compound was the most abundant component (54.96%) within the study. They evaluated the acaricidal activity of the *S. africana* Jord. & Fourr. and the *Hertia cheirifolia* (L.) Kuntze essential oils, with positive impacts upon the mortality rates of the *T. urticae* Koch and important effects in the reduction of oviposited eggs.

In another study, this same group of researchers tested the effect of the *Chrysanthemum coronarium* L. essential oil on the *T. urticae* Koch and produced mortality rates of 88% and 93% on larvae and adult females, respectively [88]. In the same year, another paper was published by Afify et al. (2012) [89], who tested the acaricidal activity of *Chamomilla recutita* L. extract on the *T. urticae* Koch. The LD_50_ values obtained for adults and eggs in this study were 0.65% and 1.17%, respectively. In this study, the authors identified the main compounds of *C. recutita* L. by means of gas chromatography–mass spectrometry. The most predominant compounds were α-bisabolol oxide (35.25%) and trans-β-farnesene (7.75%). The essential oil from the aerial part of *Achillea mellifolium* L. showed LD_50_ values of 1.208% *v*/*v* and 1.801 µL/L air when evaluated through leaf dipping and fumigation, respectively. The GC–MS chemical profile of this oil was mainly composed of piperitone (12.8%) and *p*-cymene (10.6%) [64].

However, not all studies using species from this plant family obtained satisfactory results in terms of the *T. urticae* Koch. For example, extracts obtained from *Artemisia absinthium* L.—known insecticides and acaricides used throughout the world to control aphids—demonstrated weak activity upon the *T. urticae* Koch, as reported by Aslan et al. (2005) [90]. Similarly, Derbalah et al. (2013) [91] found that the extract of castor leaves (*Artemisia cinae* O. Berg & C.F. Schmidt ex.Plajakov) exhibited low toxicity against the *T. urticae* Koch, with an LD_50_ of 1326.53 ppm. Similarly, Pavela et al. (2016) [92] studied the effect of the methanolic extract taken from leaves of the *Tithonia diversifolia* Hemsl. on *T. urticae* Koch and its ethyl acetate fraction in order to measure acute and chronic toxicity as well as its inhibitory effects on oviposition. In acute toxicity trials, mortality did not exceed 50%, even for the highest dose evaluated (150 μg/cm^3^). On the other hand, in the chronic toxicity tests on the fifth day after application, the LD_50_ of the methanolic extract was 41.3 μg/cm^3^, and the LD_90_ was 98.7 μg/cm^3^. However, the two extracts caused inhibition in the oviposition of mites.

#### 3.3.6. Family Boraginaceae

A low polar extract from roots of *Onosma visianii* Clem. demonstrated significant chronic toxicity and oviposition inhibition on *T. urticae* Koch adult females (LD_50_ = 2.6 µg/mL). Eleven naphthoquinone-type related compounds were isolated and structurally elucidated [93]. Although all isolated derivatives exhibited effects against this mite, isobutylshikonin and isovalerylshikonin were found to be the most active isolated compounds (LD_50_s = 2.69 and 1.06 µg/mL, respectively).

#### 3.3.7. Family Burseraceae

Several species belonging to this family exhibit anti-inflammatory properties [94]. They are considered to be anticarcinogenic agents with antimalarial, antidiarrheal, and antifever properties and uses as insecticides [95], antimicrobials, and antioxidants [96] (among others) for disease treatment [95]. However, some studies have pursued applications in agriculture, specifically for the management of important pests. In that respect, Pontes et al. (2007) [97] studied the acaricidal and the repellent effects of the *Protium bahianum* Daly plant resin oil on the *T. urticae* Koch by fumigant tests. For this, they kept mites in leaf discs of *Canavalia ensiformis* (L.) DC. inside 9 cm Petri dishes as test chambers. Each chamber had a strip stuck on the inner side that was saturated with different amounts and concentrations of the oil (5, 10, 15, 20, and 25 μL, corresponding to 2, 4, 6, 8, and 10 μL/L of air, respectively). They evaluated the fresh resin oil and the old resin oil separately. Results showed that the fumigant effect of the oil in both cases increased with concentration and exposure times and had mortality rates of 79.6% and 59.0% after 72 h for the old and the new resin oils, respectively. Regarding the deterrent effect of oviposition, the fresh resin oil presented an increased activity, with only 14 eggs oviposited at 72 h at a concentration of 10 μL/L of air. In repellency tests, only fresh resin oil showed positive effect against mites.

#### 3.3.8. Family Cannabaceae

Although this family of plants is recognized for its various pharmaceutical uses, little has been studied about its effects as an insecticidal and an acaricidal agent. Among the studies that have been performed, Yanar et al. (2011) [60] used the extract obtained from the flower buds of *Humulus lupulus* L. on *T. urticae* Koch adults at 5% (adhesive tape method) and at 50% (residual film method). Using the adhesive tape methodology (in which 1 mL of solution was applied to the tape left for 4 to 5 h to dry, and 20 adult females were then placed on it), the mortality rate after 24 h was 67.84% ± 2.52%. On the other hand, with the residual film methodology (in which the extract was applied to a 90 mm Petri dish, distributed homogeneously, and left for 2 to 4 h to dry before the addition of 20 *T. urticae* Koch adult females), the mortality observed after 24 h was 56.37% ± 0.99%. The acaricidal effect against this mite of an essential oil from panicles of hemp (*Cannabis sativa* L.) was also evaluated, exhibiting 83.28% of mortality on adult females at 0.10% [98].

#### 3.3.9. Family Caryophyllaceae

The acaricidal effect of an aqueous extract from roots of *Saponaria officinalis* was evaluated against all developmental stages of *T. urticae* Koch [66]. The lowest sensitivity was found for adults (LD_50_ = 0.31% w/v), while eggs revealed the highest sensitivity (LC_50_ = 1.18% w/v). Oviposition was also inhibited by this extract (LC_50_ = 0.91% w/v).

#### 3.3.10. Family Combretaceae

There are several plant species of this group on which acaricidal activity studies of the *T. urticae* Koch have been carried out—the majority of them successfully. An example of this is the study performed by Hiremath et al. (1995) [71], who compared the activity of the methanolic extracts obtained from 21 different species of African plants against adults of the *T. urticae* Koch using the leaf immersion method. Among the results found, the *Combretum micronthum* G. Don. and the *Piloitigma vetilicolin* whole plant extracts demonstrated effects on the rates of *T. urticae* Koch mortality of between 40% and 60%.

#### 3.3.11. Family Convolvulaceae

There are few studies on the *T. urticae* Koch that involve this plant family, with plants of the genera *Convolvulus* and *Ipomaea* being the most used. Chermenskaya et al. (2010) [99] studied the effect of the species *Convolvulus krauseanus* Regel. and Schmalh. on three species of pest arthropods, among which was the *T. urticae* Koch. From this study, which gathered the effect of extracts from 123 plant species, they concluded that the *C. Kraseanus* Regel. and Schmalh. roots extract was one of the two that showed the highest miticidal effect [together with the *Ailanthus altissima* (Mill.) Swingle leaf extract, Simarubaceae], causing a mortality rate of 95.6% after seven days from the application (using the immersion method).

#### 3.3.12. Family Cupressaceae

Essential oils from two plants of this family were evaluated against adult females of *T. urticae* Koch in the same study [55]. Oil from leaves of *Cupressus macrocarpa* Hartw. ex Gordon had an LD_50_ of 5.69 µL/L air, whereas *Thuja orientalis* L. leaves resulted in an LD_50_ of 7.51 µL/L air. The main compounds in these essential oils were β-citronellol (35.92%) and α-pinene (35.49%), respectively.

#### 3.3.13. Family Euphorbiaceae

The species of this family have not been well studied in terms of their pesticide properties. One of the works carried out in this area was that of Dang et al. (2010) [100], who investigated the effect of the dried root extract of *Euphorbia kansui* S.L. Liou ex S.B. Ho on the *T. urticae* Koch, as well as that of two of its compounds separately: 3-*O*-(2,3-dimethylbutanoyl)-13-ododecanoilingenol (compound 1) and 3-*O*-(2′*E*, 4′*Z*-decadienoyl)-ingenol (compound 2). Concerning the extract, they found that it generated mortality rates of 27% and 55% at concentrations of 3 and 5 g/L, respectively. When testing the two compounds obtained by fractionation and evaluating them on mites, they determined that compound 1 caused mortality rates of 45% and 59% when applied at 500 and 1000 mg/L, respectively. In contrast, compound 2 showed no acaricidal activity during the study.

On the other hand, in 2015, Numa et al. (2015) [61] published a study in which they tested the susceptibility of *T. urticae* Koch females to the *Cnidoscolus aconitifolius* (Mill) I.M. Johnst. leaf extract using the leaf immersion methodology merged with direct application using an airbrush. In this study, they determined that a dose of 2000 μg/mL was the only one that did not show differences in the positive control (based on chlorfenapyr as the active ingredient). This dose could be the most appropriate for an extract formulation based of this plant during its potential use in the control of pests in agricultural crops, taking into account the fact that it caused a 92% rate of mortality of mite females in the trials.

#### 3.3.14. Family Fabaceae

This family is well known as an aspect of human diets throughout the world. Several studies have been carried out to evaluate the effects of their plant extracts on arthropods with very varied results. These include the study performed by Hiremath et al. (1995) [71], who compared the activity of methanolic extracts obtained from 21 different species of African plants against adults of the *T. urticae* Koch using the leaf immersion method. The most active extracts were those obtained from the leaves, the fruits, and the whole plant of *Prosopis chinensis* (Molina) Stuntz, which caused mortality rates between 61% and 80% for the leaf extract and higher than 80% in the case of the extracts obtained from the fruits and the whole plant. The plant oil of *Millettia pinnata* L. showed an LD_50_ of 0.004% on adult females after four days of testing [101].

#### 3.3.15. Family Gramineae (Poaceae)

Although this family is made up of nearly 10,000 plant species, studies involving the effect of its plant extracts on the *T. urticae* Koch have focused on only some of the 55 species that make up the *Cymbopogon* genus [102]. In one of these cases, Choi et al. (2004) [42] included the oil from *Cymbopogon nardus* (L) Rendle within the 53 essential oils that they evaluated on the *T. urticae* Koch. This oil showed a positive result, causing a mortality rate greater than 90% on adults of this phytophagous mite. In a study of another species of genus *Cymbopogon*, Han et al. (2010) [68] examined the effect of Citronella Java oil on the *T. urticae* Koch, evaluating its fumigant effect. To do this, they took disc-shaped bean leaves and placed them on moistened cotton contained in Petri dishes together with *T. urticae* Koch adult mites. On each Petri dish, a mesh cover was placed and placed over this was filter paper moistened with the essential oil. Under these conditions, the LD_50_ found was 22.5 μg/cm^3^.

#### 3.3.16. Family Lamiaceae

The effects of plant extracts and essential oils from the species that make up this family have been the most studied on the phytophagous mite *T. urticae* Koch. Among the studies reported in the literature are, for instance, those from the species *Rosmarinus officinalis* L. and *Salvia officinalis* L. The essential oils of these plants demonstrated effective control over populations of the *T. urticae* Koch and a decrease in the number of oviposited eggs when concentrations increased [53]. In a similar way, Choi et al. (2004) [42] performed trials using the *S. officinalis* L. essential oil on the same species of mite, obtaining an adult mortality rate of 82%. In the same study, they included another species from the family Lamiaceae—*Mentha spicata* L.—from which they obtained the essential oil that was evaluated on the *T. urticae* Koch. As a result, the mortality rate of these arthropods in the adult stage was 81%.

On the other hand, Rasikari et al. (2005) [103] carried out a screening of the leaf extracts of 67 species of plants belonging to the Lamiaceae family. They were evaluated on the *T. urticae* Koch, which were applied by direct contact with the Potter Tower to bean leaves kept in Petri dishes with cotton. From the extracts tested, 14 had a moderate to acute toxic effect on mites. From these, extracts obtained from the plants *Clerodendrum traceyi* F. Muell., *Premna serratifolia* L., *Ceratanthus longicornis* (F.Muell.) G. Taylor, *Plectranthus habrophyllus* P.I. Forst, and *Plectranthus* sp. Hann caused a 100% mortality rate, whereas the extracts of *Gmelina leichardtii* F.Muell. & Benth, *Premna acuminata* R. Br., *Viticipremna queenslandica* Munir, *Plectranthus diversus* S.T. Blake, *Plectranthus glabriflorus* P.I. Forst, and *Plectranthus suaveolens* S.T. Blake caused mortality rates that were between 90% and 99%.

In 2006, a study performed by Miresmailli et al. (2006) [104] was published. In that investigation, they tested the effect of the *R. officinalis* L. essential oil on the *T. urticae* Koch. For that, they took two different populations of mites, one from bean plants and another from tomato plants. For the tests, they used five different concentrations (2.5, 5, 10, 20, 40, and 80 mL/L) of the essential oil diluted in methanol and water (70:30 *v*/*v*). In order to evaluate the mortality rates of mites, they took 3 mm disc leaves within Petri dishes, to which they applied 20 μL of the treatment solution. Once dried at room temperature, they placed five adult females on the leaves and kept them at a temperature of 26 ± 2 °C, a relative humidity (RH) between 55% and 60%, and a photoperiod of 16:8 (light:dark). From these assays, they determined that the LC_50_ for the females maintained on bean plants was 10 mL/L, while for the females kept on tomato plants, it was 13 mL/L. Moreover, with a concentration of 20 mL/L, a mortality of 100% of females produced in bean plants was obtained, whereas a 40 mL/L concentration was necessary before females on the tomato plants reached total mortality (100%).

Additionally, Miresmailli et al. [104] identified the components of *R. officinalis* L. essential oil using GC–MS by column chromatography and tested them individually on the *T. urticae* Koch. In the case of mites reared on bean plants, two compounds revealed a significant toxicity—1,8-cineol and α-pinene (with 88% ± 4.8% and 32% ± 4.8% mortality, respectively)—whereas for mites raised on tomato plants, the same two compounds were those that revealed a significant toxicity. The resulting values were 80% ± 6.2% and 72% ± 4.8% for 1,8-cineol and α-pinene, respectively.

In a similar study, Çalmaşur et al. (2006) [105] tested the effect of the vapors of three essential oils from *Micromeria fruticosa* L., *Nepeta racemosa* L., and *Origanum vulgare* L. on nymphs and adults of the *T. urticae* Koch and adults of the *Bemisia tabaci* Gennadius, finding the highest mortality rates (96.7%, 95%, and 95%, respectively, for *T. urticae* Koch, and 100% for *B*. *tabaci* Gennadius) when using doses of 2 μL/L of air at 12 h of exposure. Han et al. (2010) [68] also studied several essential oils obtained from species of this family. To do this, they evaluated its fumigant effects on the *T. urticae* Koch and, as a result, obtained LD_50_s of 22.7, 22.8, 23.7, 38.8, 39.5, and 63.7 μg/cm^3^ for *Thymus vulgaris* L., *Mentha* L. *piperita*, *Mentha pulegium* L., *Mentha spicata* L., *Ocimum basilicum* L., and *Salvia officinalis* L., respectively.

In 2012, Afify et al. (2012) [89] tested the acaricidal activity of *Majorana hortensis* Moench extract on the *T. urticae* Koch. The LD_50_ values obtained for adults and eggs in the trial were 1.84% and 6.26%, respectively. In the study, they identified the main compounds of *M. hortensis* Moench by means of gas chromatography–mass spectrometry as terpinen-4-ol (23.86%), *p*-cymene (23.40%), and sabinene (10.90%)—the main compounds for this species. In the same year, Attia et al. (2012) [88] tested the effect of the essential oil of *Mentha pulegium* L. on the *T. urticae* Koch, obtaining a mortality rate of 91% in larvae and adult females. The same essential oil was evaluated by Choi et al. (2004) [42] on the same mite species, in which a mortality rate higher than 90% was obtained. Within the same experiment, they analyzed the effect of the essential oil of the *Mentha piperita* L., in which the mortality rate also exceeded 90%. On the other hand, Amizadeh et al. (2013) [84] studied the fumigant effect of the essential oil obtained from leaves of the *Satureja sahendica* Bornm. on eggs and adult females. The LD_50_ obtained for females was 0.98 μL/L, while it was of 0.54 μL/L for eggs.

#### 3.3.17. Family Meliaceae

The insecticidal properties of plants belonging to the family Meliaceae have been studied extensively [106]. For this reason, Ismail (1997) [107] evaluated the relative toxicity of the extracts of *Melia azedarach* L. and some synthetic acaricides against recently hatched larvae of the *T. urticae* Koch and third-instar larvae of the predatory beetle, *Stethorus gilvifrons* Mulsant. The methanolic extract of the plant was the most effective among the tested products, followed by the extracts of acetone and petroleum ether. The toxicity of the plant material obtained was less active against the predator compared to the effect it had on the two-spotted spider mite, in which a decrease in fecundity was also observed. The study of the joint action of the products also revealed a strong synergy in the bromopropylate mixture with the methanolic extract of the *M. azedarach* L. Interestingly, this mixture demonstrated no effect on the predator.

In a similar way, Brito et al. (2006) [37] tested the toxicity of different commercial products based on one of the plants with the highest pesticide potential, the Neem (*Azadirachta indica* A. Juss.). It was tested not only on the *T. urticae* Koch but also on its predators, *Euseius alatus* DeLeon and *Phytoseiulus macropilis* Banks. In this study, they found that the formulation of the product Neemseto (1%) was the one that obtained the best result on the *T. urticae* Koch by topical contact. In the same way, they tested the product at different concentrations (0.25%, 0.5%, and 1.0%) and found that the product had a repellent effect on *T. urticae* Koch and *E. alatus* DeLeon; however, it did not affect the *P. macropilis* Banks. Additionally, the Neemseto exhibited an important reduction in *T. urticae* Koch fecundity, but on the predatory mites, a significant decrease was only observed when mites were exposed to the highest concentrations. This shows that this product can be a promising option for the management of the two-spotted spider mites within integrated pest management schemes given its relative compatibility with predatory mites.

#### 3.3.18. Family Myrtaceae

*T. urticae* Koch toxicity studies involving these plants have had varying results. First, Choi et al. (2004) [42] determined that the *Eucalyptus citriodora* Hook’s essential oil is capable of causing a mortality rate of more than 90% on *T. urticae* Koch adults. This essential oil was also tested by Han et al. (2010) [68] on the same mite species using the vapor-phase mortality bioassay; they found similar fumigant activity results to those obtained previously [42]. The test performed consisted of placing 3 cm diameter bean leaf discs on wet cotton inside Petri dishes, each with 20 adult mite individuals [68]. On each Petri dish, they installed a mesh cover on which a filter paper moistened with the essential oil at the evaluated concentrations was placed (after drying for two minutes). From these experiments, they estimated an LD_50_ of 19.3 μg/cm^3^.

On the other hand, they also wanted to evaluate the fumigant effect of *Syzygium aromaticum* (L.) Merr. & L.M. Perry essential oil. Within the study, they found an LD_50_ value of 23.6 μg/cm^3^ on *T. urticae* Koch adults. In 2011, Afify et al. [108] tested the activity of six extracts of *Syzygium cumini* (L.) Skeels at three different concentrations (75, 150, and 300 μg/mL) on the *T. urticae* Koch. The highest mortality rates were obtained with the ethanolic extract (98.5%), followed by the hexane extract (94%) and the ether-ethyl acetate extract (90%). The LD_50_ values obtained were 85, 101, 102, and 98 μg/mL, respectively. The same group of researchers in 2012 conducted a study to measure the acaricidal activity of *Eucalyptus* sp. on the same mite [89]. The LD_50_ values obtained for adults and eggs in the assay were 2.18 and 7.33 μg/mL, respectively.

In 2013, Amizadeh et al. [84] also tested the fumigant effect of some essential oils of this family, including those obtained from leaves and fruits of the *Eucalyptus microtheca* F. Muell. on both eggs and adult females of the *T. urticae* Koch. For the tests, mites were placed on bean leaf discs laid in plastic containers in which an oil-impregnated filter paper was held without coming into direct contact with leaf discs or mites. The LD_50_s on the adult females were 1.52 μL/L and 5.7 μL/L for the extracts of leaves and fruits, respectively, while for eggs, they were 0.56 μL/L and 2.36 μL/L for leaf and fruit extracts, respectively.

#### 3.3.19. Piperaceae Family

There have been few studies carried out concerning the effects of extracts of species of the Piperaceae family on the *T. urticae* Koch—particularly considering the fact that they have focused on very few species of the genus *Piper*, which has more than 1000 species [109]. One of those studies was developed by Araújo et al. (2012) [110], who reported acaricidal and repellent activity of the essential oils obtained from *Piper aduncum* L. leaves and its components separately on the *T. urticae* Koch. The repellent activity was attributed to the components (*E*)-nerolidol, α-humulene, and β-caryophyllene, while the toxicity was attributed to β-caryophyllene. The extracts and their components exhibited a better performance in fumigation than in contact.

#### 3.3.20. Family Ranunculaceae

In general terms, the toxicity studies of extracts of these plants used on the *T. urticae* Koch have not been very satisfactory. A case demonstrating this is the study conducted by Derbalah et al. (2013) [91], which found that the black cumin seeds (*Nigella sativum* L.) extract showed a low toxic effect on the *T. urticae* Koch, with an LD_50_ of 708.57 ppm. However, some species of this family—such as *Aconitum soongaricum* Stapf and *Clematis orientalis* L.—have shown toxic effects on the *T. urticae* Koch with mortality rates ranging between 50% and 80% of mites [99].

#### 3.3.21. Family Rutaceae

In 2005, Tewary et al. [111] tested two concentrations (5000 and 10,000 ppm) of the *Zanthoxylum armatum* DC. leaf extract on the arthropods *H. armigera* Hübner, *P. xylostella* L., *T. urticae* Koch, and *A. craccivora* Koch, with mortality rates of 46% at 10,000 ppm in the *H. armigera* Hübner, 42% at 10,000 ppm in the *P. xylostella* L., 36% and 39% at 5000 and 10,000 ppm, respectively, in the *T. urticae* Koch, and 30% and 65% at 5000 and 10,000 ppm, respectively, in the *A. craccivora* Koch. On the other hand, Attia et al. (2012) [88] also included plants of the Rutaceae family, since they proved the effect caused by the essential oil of *Haplophyllum tuberculatum* (Forssk.) A. Juss. on the *T. urticae* Koch, obtaining a mortality of 93%.

Da Camara et al. (2015) [67] demonstrated that essential oils obtained from the epicarp of pear orange fruits (*Citrus sinensis* Osbeck var. Pera) and the lime orange (*Citrus aurantium* L.) had repellent effects against the *T. urticae* Koch, with very similar repellency results to those obtained with eugenol. Using mass spectrometry, 27 compounds were idenitified both in *C. sinensis* Osbeck and in *C. aurantium* L., which corresponded to 98.1% and 98.9% of the total constituents of the two extracts, respectively. This demonstrated that the major compound in the two essential oils was d-limonene. Within this study, the authors determined that all the identified compounds were responsible for the repellency.

#### 3.3.22. Family Santalaceae

Within this family, Roh et al. (2011) [112] studied the effect of *Santalum* L. sp. essential oil on the *T. urticae* Koch using the leaf immersion method. Through this methodology, they found that the mortality rate of mites was 87.2% ± 2.9%. Additionally, they noticed an oviposition decrease of 89.3% on leaves treated with oil. Subsequently, they evaluated a mixture of α and β–Sandalool—the two main compounds of *Santalum* L. sp.—on the *T. urticae* Koch and obtained a mortality of 85.5% ± 2.9% and a decrease of 94.7% in fecundity.

#### 3.3.23. Family Scrophulariaceae

The toxic effects of Scrophulariaceae plants on the *T. urticae* Koch have been less studied than plant species of other groups. Within the investigations carried out in this regard, Khambay et al. (1999) [113] studied the effect of two compounds of *Calceolaria andina* Benth extract with recognized insecticidal activity—2-(1,1-dimethylprop-2-enyl)-3-hydroxy-1,4-naphthoquinone (compound **1**) and 2-acetoxy-3-(1,1-dimethylprop-2-enyl)-1,4-naphthoquinone (compound **2**)—on 29 pest species, including the *T. urticae* Koch. The LD_50_s for this species were 80 ppm and 30 ppm for each compound, respectively. The two cases were evaluated using the micro-immersion method. Additionally, they performed the same test on individuals from a population that showed resistance to chlorpyrifos and bifenthrin using the same compounds of *C. andina* Benth extract, thus obtaining LD_50_s of 44 ppm and 33 ppm for compounds **1** and **2**, respectively.

#### 3.3.24. Family Simarubaceae

The toxicity of plant extracts from species of this family on the tetraniquid mite *T. urticae* Koch have not been well studied. Among the studies accomplished, Latif et al. (2000) [114] tested the extract from *Quassia* sp. aerial parts on this mite at a concentration of 10,000 ppm, finding acaricidal activity. Subsequently, they identified the quassinoid Chaparinone compound and tested it separately, obtaining an LC_50_ of 47 ppm. Chermenskaya et al. (2010) [99] evaluated extracts from 123 different plant species on the *T. urticae* Koch, *Frankliniella occidentalis* Pergande and *Shizaphis graminum* Rondani, using the leaf immersion method. Within these extracts, one that demonstrated a high acaricidal effect was obtained from the *Ailanthus altissima* (Mill.) Swingle leaves, which caused a mortality rate of 97.4% after 7 days of evaluation.

#### 3.3.25. Family Solanaceae

Although most studies involving plant extracts tested on the *T. urticae* Koch have focused on assessing the effects on mortality and fecundity, those involving the Solanaceae family have been mostly dedicated to determining the repellent effects of certain extracts. Such is the case of the study conducted by Snyder et al. (1993) [115]. They isolated dihydrofarnesoic acid as one of the phytoconstituents in trichomes of *Lycopersicon hirsutum* Dunal, and its repellent effect on the phytophagous mite was then evaluated. For this purpose, 10 μL of the extract was applied to a filter paper separated by 1.5 cm from another similar filter, which was impregnated with 10 μL of hexane. Once the solvent was evaporated, a strip of filter paper was positioned to connect the two filter papers, and a mite was placed in the middle of the paper bridge to evaluate its displacement preference. This process was performed with approximately 40 adult females. According to the obtained results, they concluded that dihydrofarnesoic acid exhibited a repellent activity against the *T. urticae* Koch. Similarly, Antonious et al. (2006) [116] also evaluated toxic and repellent effect of the fruit extracts of *Capsicum chinense* Jacq., *Capsicum frutescens* L., *Capsicum baccatum* L., *Capsicum annuum* L., and *Capsicum pubescens* Ruíz & Pav. In their results, they determined that the highest mortality rate (45%) occurred when using the extract of the *C. annuum* L., while the extracts of the fruits of the *C. baccatum* L. and *C. annuum* L. caused repellence on mites.

Extracts of leaves and seeds of the *Datura stramonium* L. were used by Kumral et al. (2009) [5] to evaluate their acaricidal, repelling, and deterrent effects on oviposition over *T. urticae* Koch adults at 167.25 mg/L and 145.75 mg/L (for leaves and seeds, respectively). For these tests, they used a Potter Tower in order to place the mites on leaf discs contained in Petri dishes. These concentrations caused 98% and 25% of the mortality, respectively, for the two concentrations after 48 h of application. Through a simple logistic regression analysis, they determined that an increase in the leaf extract dose caused a significant increase in mite mortality, while the effect of increasing the dose of the seed extract was not significant. Based on Probit analysis, they estimated that the lethal dose (LD_50_) with the leaf extract was 70.59 mg/L. According to the Pearson X^2^ test, they concluded that mites showed a strong tendency to flee from areas treated with leaf and seed extracts to untreated areas.

#### 3.3.26. Verbenaceae Family

In this family, a highlighted study was conducted by Cavalcanti et al. (2010) [117], in which they carried out a characterization of the essential oils of the *Lippia sidoides* Cham. (Verbenaceae) by GC–MS and tested their acaricidal activity on *T. urticae* Koch females. They concluded that the compounds thymol and carvacrol—as well as the essential oil of *L. sidoides* Cham.—showed a promising miticidal activity against this mite.

### 3.4. Additional Studies with Isolated Compounds Obtained after Plant Extract Fractionation

As with essential oils and plant extracts, a considerable number of their isolated constituents have also been tested on the *T. urticae* Koch. For example, Lee et al. (1997) [118] studied the insecticidal and the acaricidal effects of several monoterpenes and their possible phytotoxicity in maize plants that served as hosts of the *Diabrotica virgifera virgifera* LeConte, *T. urticae* Koch, and *Musca domestica* L. Twenty-nine compounds belonging to different chemical classes were tested against the *T. urticae* Koch by means of the leaf immersion method.

These tests used: the alcohols carveol, carvomentenol, citronellol, geraniol, 10-hydroxygeranol, isopulegol, linalool, menthol, perilyl alcohol, aterpineol, and verbenol; the phenols carvacrol, eugenol, and thymol; the ketones (−)-carvone, (+)-carvone, (+)-fenchone, menthone, pulegone, tuyone, and verbenone; the aldehydes citral and citronellal; citronelic acid; ether 1,8-cineol; and the hydrocarbons limonene, α-terpinene, and y-terpinene.

All compounds were tested in water with Triton X-100 as a wetting agent at 10,000 and 1000 ppm, and the activity was evaluated 24, 48, and 72 h after the treatment. The toxicity varied depending on the concentrations and the exposure times. All of the monoterpenes tested—except for 1,8-cineole, 10-hydroxygeraniol, aterpineol, verbenol, and verbenone—caused a 100% mortality rate at the highest concentration after 24 h. However, carvacrol was the most effective compound in the lowest concentrations, followed by citronellol.

On the other hand, geraniol produced a 100% rate of mortality, while its 10-hydroxy geraniol analogue exhibited a 0% mortality rate. During the trial, a longer exposure time increased acaricidal effects. Alternately, the most effective monoterpenoids (carvacrol, carvomenthenol, carvone, citronellol, eugenol, geraniol, perilyl alcohol, 4-terpineol, thymol) were evaluated separately in more detailed tests. From these compounds, carvomentenol and 4-terpineol demonstrated greater acaricidal activity (LC_50_s = 59 and 96 ppm, respectively).

In another study, Martínez et al. (2005) [119] examined the effect of azadirachtin at 64 and 128 ppm on different biological parameters of the *T. urticae* Koch, such as longevity, fecundity, fertility, and offspring development. The tests were performed on bean leaf discs in Petri dishes using the Potter Tower. The results found that this compound affected mortality and fecundity but exhibited no effects on fertility and offspring development. In a later analysis of life table, they determined that, with the application of azadirachtin at 80 ppm, the adult survival rate was reduced to 50%. Duso et al. (2008) [34] also tested the toxicity of Azadirachtin on the *T. urticae* Koch. In that case, the micro-immersion bioassay methodology was implemented using a concentration of 4.5 g of active ingredient/L on *T. urticae* Koch females. For those conditions, the mortality rate obtained was 86.49%.

Similarly, Han et al. (2011) [120] tested some constituent compounds of the *Eucalyptus citriodora* Hook extract and other plants on resistant and susceptible acaricidal *T. urticae* Koch females. Among them, those that showed the highest toxicity were menthol (LD_50_ of 12.9 μg/cm^3^) and citronellium acetate (LD_50_ of 16.8 μg/cm^3^), evaluated on females susceptible to acaricides. Other compounds such as β-citronellol, citral, geranyl acetate, and eugenol also demonstrated a high toxic activity, with LD_50_s between 21.7 μg/cm^3^ and 24.6 μg/cm^3^. When comparing the mortality results obtained for both susceptible and acaricide-resistant mites, the researchers estimated that they were very similar to each other and therefore evidenced that the mechanisms of action of the components of the essential oil and of the synthetic acaricides are different and do not present processes that promote cross-resistance.

One year later, Akhtar et al. (2012) [121] studied the effect of eight quinones on the *T. urticae* Koch—*Myzus persicae* Sulzer, *Myzocallis walshii* Monell, and *Illinoia liriodendri* Monell—using the leaf immersion method. The compound plumbagine was the one that exhibited the greatest activity on the mite, with an LC_50_ of 0.001%. Marčić and Međo (2014) [122] also performed experiments with secondary metabolites from plants. In their study, they tested a combination of oximatrin and psoralen (0.2% and 0.4%, respectively) on the *T. urticae* Koch and measured acute toxicity and repellency. The applications were made on bean leaves with a Potter Tower, and the subsequently calculated LD_50_s were 55.49, 52.68, 6.88, 13.03, and 8.8 μL/L for eggs, females that had not oviposited, larvae, protonymphs, and deutonymphs, respectively. Additionally, they noticed that, in preferential tests on the leaves, the mites tended to be located in the middle of the untreated leaf, at which point the oviposition was greater.

The same authors also tested compounds from the Neem extract (azadirachtin-A) on females of the two-spotted spider mite [123]. For this case, they introduced bean leaf discs inside Petri dishes with moistened cotton and made applications of the product using a Potter Tower in the middle of the leaf. They concluded that females preferred to be located in the middle of the leaf not treated with the product and, in the same way, they observed that oviposition was higher in females that were located in the untreated areas.

## 4. Conclusions

In conclusion, 458 records of plant species from 67 plant families (listed in this survey) have repellent or acaricidal effects against the *T. urticae* Koch under laboratory conditions. The efficacy is available at different levels depending on species, extractions (extract or essential oils), plant parts used, and concentrations of test extract/essential oil. Among the most studied botanical families for this purpose are plants from Lamiaceae, Asteraceae, Myrtaceae, and Apiaceae taxons. Extracts from species including *Celosia Trygina* L., *Cassia mimosoides* L., *Clome viscosa* L., *Boscia senagalensis* (Pers.) Lam. Ex. Poir., *Cobretum micranthum* G. Don, *Ipomaea asarifolia* (Desr.) Roem. and Schult., *Cnidoscolus aconitifolius* (Mill) I.M. Johnst., *Azadirachta indica* A. Juss., *Syzygium cumini* (L.) Skeels, *Papaver rhoeas* L., *Plantago major* L., *Ailanthus altissima* (Mill.) Swingle, and *Capsicum annuum* L. exhibited better acaricidal properties with efficacies between 90% and 100% at a concentration range between 0.2% and 1%—comparable to some commercial acaricides. LD_50_ values can be found below 20 µg/mL or 5 µL/L air. Thus, botanical-based preparations can be a good source of effective acaricidal preparations either as extracts or as essential oils. Although the information herein presented only concerns a basic screening of the acaricidal efficacy of botanicals at laboratory (in vitro) levels, several plants could be considered for future research on field evaluations or as sources of acaricide compounds. In this sense, several compounds such as azadirachtin, 10-hydroxygeraniol, terpineols, verbenol, verbenone, carvacrol, plumbagine, linalool, and citral, among others, have been isolated as bioactive acaricidal compounds. In future studies, attention may be focused on acaricidal activity rather than on repellent properties to facilitate two-spotted mite control. However, formulations and application rates similar to those used by farmers must be assessed in order to achieve more predictive results in further field experiments. Sublethal effects must also be relevant in future research, since those effects could produce other subsequent problems or benefits in the control of mites. Finally, more compatibility studies and phytotoxicity as well as extract stability, extraction standardization, and field formulations are required to ensure good results on integrated pest management programs for *T. urticae* Koch control using effective botanicals.

## Figures and Tables

**Table 1:** The resistance of different populations of *T. urticae* Koch—from the state of Pernambuco (Brazil)—to 10 different acaricides. Adapted from Ferreira et al. [14].

Acaricide	Population	N°	LC_50_ (mg/L) *	LC_95_ (mg/L) *	RR_50_
Diafenthiuron	Petrolina II	426	6.6	70	1
	Piracicaba	484	10.7	105	1.6
	Brejão	340	4053	93,708	619
	Bonito	401	7732	133,440	1180
Milbemectin	Piracicaba	484	0.6	8.3	1
	Petrolina II	455	5.4	101	9.9
	Bonito	373	357	3726	650
	Brejão	380	384	2386	700
Fenpyroximate	Piracicaba	315	22	341	1
	Petrolina II	469	87	1929	4
	Bonito	378	3246	10,014	200
	Brejão	387	4343	16.234	150
Clorfenapyr	Piracicaba	424	1.3	9.3	1
	Petrolina II	481	2.8	20.1	2.2
	Brejão	477	735	4157	570
	Bonito	524	4652	94.598	3600
Spirodiclofen	Piracicaba	401	16.4	1590	1
	Petrolina II	547	37.5	370.870	2.3
	Bonito	538	6401	127.750	390
	Brejão	414	6586	56.390	400
Fenbutatin oxide	Piracicaba	465	0.83	436	1
	Petrolina II	538	1.72	1093	2.1
	Bonito	477	293	52.892	350
	Brejão	459	1705	197.990	2048
Propargite	Piracicaba	472	6.5	28	1
	Petrolina II	397	15	66	2.3
	Bonito	395	291	990	45
	Brejão	391	622	4410	96
Hexythiazox	Piracicaba	416	2938	64.871	1
	Petrolina II	404	4370	100.510	1.5
	Brejão	440	12,700	605.400	4.3
	Bonito	415	1384	381.630	4.7
Spiromesifen	Piracicaba	418	373	18.404	1
	Petrolina II	487	487	17.752	1
	Brejão	467	1388	42.781	3.7
	Bonito	470	3201	90.424	8.6
Abamectin	Petrolina II	613	0.0011	0.033	1
	Piracicaba	714	0.0084	0.066	8
	Petrolina I	676	0.036	0.205	34.4
	Gravatá	787	0.041	1.66	39.3
	Goiânia	584	1.79	27.9	1716
	Brejão	610	118	3000	113.532
	Bonito	693	326	3397	295.270

* LC_50_: the mortality-causing concentration of 50% of the test population. LC_95_: the mortality-causing concentration of 95% of the test population. N°: the number of mites used in the trial. RR_50_: the resistance proportion between the resistant population and the susceptible one at LC_50_.

**Table 2:** A list of pest arthropods based on the reported number of active ingredients resistance and the number of reported cases per species—adapted from Van Leeuwen et al. (2010, 2012) [22,23]. The information for the species *Plutella xylostella* L., *Myzus persicae* Sulzer, *Leptinotarsa decemlineata* Say, *Blatella germanica* L., and *Panonychus ulmi* Koch correspond to the cases reported up to 2010.

Species	Taxonomy	Kind of Pest	Number of Active Ingredients	Cases of Resistance
*Tetranychus urticae* Koch	Acari: Tetranychidae	Crop	93	389
*Plutella xylostella* L.	Lepidoptera: Plutellidae	Crop	81	437
*Myzus persicae* Sulzer	Hemiptera: Aphididae	Crop	73	320
*Leptinotarsa decemlineata* Say	Coleoptera: Chrysomelidae	Crop	51	188
*Musca domestica* L.	Diptera: Muscidae	Urban	53	266
*Blatella germanica* L.	Blattodea: Blatellidae	Urban	43	213
*Rhipicephalus microplus* Canestrini	Acari: Ixodidae	Cattle	43	158
*Helicoverpa armigera* Hubner	Lepidoptera: Noctuidae	Crop	43	639
*Bemisia tabaci* Gennadius	Hemiptera: Aleyrodidae	Crop	45	428
*Panonychus ulmi* Koch	Acari: Tetranychidae	Crop	42	181
*Varroa destructor* Anderson y trueman	Acari: Varroidae	Bees parasite	2	10
*Ixodes scapularis* Say	Acari: Ixodidae	Cattle	0	0
*Culex pipiens* L.	Diptera: Culicidae	Disease vector	36	161
*Culex quinquefasciatus* Say	Diptera: Culicidae	Disease vector	32	256
*Tribolium castaneum* Herbst	Coleoptera: Tenebrionidae	Stored-grain pest	32	113
*Aedes egypti egypti* L.	Diptera: Culicidae	Disease vector	24	267
*Spodoptera frugiperda* Smith	Lepidoptera: Noctuidae	Crop	16	25
*Pediculus humanus* L.	Phthiraptera: Pediculidae	Disease vector	9	59
*Anopheles gambiae* Giles	Diptera: Culicidae	Disease vector	3	39
*Manduca sexta* L.	Lepidoptera: Sphingidae	Crop	3	4
*Rhodnius prolixus* Stal	Hemiptera: Reduviidae	Disease vector	3	3
*Anopheles darlingi* Root	Diptera: Culicidae	Disease vector	1	2
*Linepithema humile* Mayr	Hymenoptera: Formicidae	Urban	2	2

## Appendix A

**Table A1:** Compilation of reported studies using plant extracts and essential oils against *T. urticae* Koch under laboratory conditions.

Family	Plant Species	Source	Concentration	Bioassay*^a^*	*T. urticae* Koch Stage	Effect on *T. urticae* Koch	Identified Compounds	Ref.
Amaranthaceae	*Amaranthus viridis* L.	Whole plant extract	5000 ppm	G	Adults	Mortality between 40 and 60%	-	[108]
Amaranthaceae	*Amaranthus viridis* L.	Whole plant extract	2500 ppm	G	Adults	Mortality between 40 and 60%	-	[108]
Amaranthaceae	*Blepharis linariifolia* Pers.	Whole plant extract	5000 ppm	G	Adults	Mortality between 61 and 80%	-	[108]
Amaranthaceae	*Blepharis linariifolia* Pers.	Whole plant extract	2500 ppm	G	Adults	Mortality between 61 and 80%	-	[108]
Amaranthaceae	*Blepharis* sp.	Whole plant extract	5000 ppm	G	Adults	More than 80% of mortality	-	[108]
Amaranthaceae	*Blepharis* sp.	Whole plant extract	2500 ppm	G	Adults	Mortality between 61 and 80%	-	[108]
Amaranthaceae	*Celosia Trygina* L.	Whole plant extract	5000 ppm	G	Adults	More than 80% of mortality	-	[108]
Amaranthaceae	*Celosia Trygina* L.	Whole plant extract	2500 ppm	G	Adults	More than 80% of mortality	-	[108]
Amaranthaceae	*Chenopodium ambrosioides* Mosyakin et Clemants	Emulsifiable Concentrate	0.50%	A,C	Adults and eggs	94.7% of mortality	-	[41]
Amaranthaceae	*Chenopodium quinoa* Willd.	Seeds extract	6–9% w/v [1.24% w/v (LD_50_)]	E,F	Adult females and nymphs	Mortalities ranged from 30% to 99%	-	[89]
Amaranthaceae	*Kochia scoparia* (L.) Schrad.	-	98.13% (chloroform extraction)	A,E,H	Adult females	92.58% of mortality	-	[52]
Amaryllidaceae	*Allium cepa* L.	Essential oil	-	D	Larvae and adults.	Mortalities of 65% (larvae) and 67% (adults)	-	[88]
Amaryllidaceae	*Allium cepa* L.	Peel fruit extract	1%	G	Adult females	Mortality between 0 and 20%	-	[99]
Amaryllidaceae	*Allium galanthum* Kar. & Kir.	Whole plant extract	1%	G	Adult females	Mortality between 20 and 50%	-	[99]
Amaryllidaceae	*Allium obliquum* L.	Whole plant extract	1%	G	Adult females	Mortality between 50 and 80%	-	[99]
Amaryllidaceae	*Allium sativum* L.	-	7.2 g/L	A,G	Adult females	LD_50_	-	[78]
Amaryllidaceae	*Allium sativum* L.	Bulb extract	7.49 and 13.5 mg/L	E,F	Adult females	LD_50_ and LD_90_ (respectively)	-	[77]
Amaryllidaceae	*Allium sativum* L.	Essential oil	-	D	Larvae and adults	Mortalities of 86% (larvae) and 61% (adults)	-	[88]
Amaryllidaceae	*Pancratium maritimum* L.	Alkaloidal ethanolic extract and bulb essential oil	0.2%. 0.36% and 1.5% respectively		-	LD_50_	-	[76]
Amaryllidaceae	*Ungernia severtzovii* Regel	Root extract	1%	G	Adult females	Mortality between 20 and 50%	-	[99]
Anacardiaceae	*Cotinus coggygria* Scop.	Essential oil	-	D	Larvae and adults	Mortalities of 58% (larvae) and 58% (adults)	-	[88]
Anacardiaceae	*Cotinus coggygria* Scop.	Aerial part extract	1%	G	Adult females	Mortality between 0 and 20%	-	[99]
Anacardiaceae	*Pistacia lentiscus* L.	Essential oil	-	D	Larvae and adults	Mortalities of 22% (larvae) and 23% (adults)	-	[88]
Annonaceae	*Annona glabra* L.	Seed extract	1000 ppm	D,G	Eggs	No effects	-	[80]
Annonaceae	*Cananga odorata* (Lam.) Hook.F. & Thomson	Essential oil	0.1%	G	Adult females	24.2% of mortality	-	[112]
Annonaceae	*Xilopia sericea* A.St.-Hill.	Leaves and fruits essential oils	4.08 µL/L	C	Adult females	LD_50_	α-pinene (0.41% leaves, 17.18% fruits), β-pinene (45.59% fruits), cubenol (57.43% leaves), myrcene (9.13% fruits), between others	[44]
Apiaceae	*Ammi visnaga*	Seed extract	17 µg/cm^2^	D,I	Eggs	LD_50_	Kheline and visnagine	[65]
Apiaceae	*Carum carvi* L.	Essential oil	19 × 10^−3^ µL/mL of air.	J	Adults	100% of mortality	-	[42]
Apiaceae	*Carum carvi* L.	Essential oil	22.4 µg/cm^3^	C,J	Adults	LD_50_	-	[68]
Apiaceae	*Carum carvi* L.	Essential oils mixed with Fatty acid potassium salts	570 ppm of essential oil and 2478 ppm of potassium salts	E	Adults	83.4% of mortality	-	[83]
Apiaceae	*Conium maculatum* L.	Flowers and leaves extract	10–50%	A,B	Adult females	Mortalities of 95.18% and 81.11%, respectively	-	[60]
Apiaceae	*Coriandrum sativum* L.	Essential oil	19 × 10^−3^ µL/mL of air	J	Adults	92% of mortality	-	[42]
Apiaceae	*Cuminum cyminum* L.	Essential oil from seeds	3.74 µL/L air	C,J	Adult females	LD_50_	α-pinene (29.1%), limonene (22%), 1,8-cineole (17.9%)	[85]
Apiaceae	*Daucus carota* L.	Essential oil	-	D	Larvae and adults	Mortalities of 5% (larvae) and 3% (adults)	-	[88]
Apiaceae	*Deverra scoparia* Coss. & Durieu	Essential oil	1.79 and 3.2 mg/L	E	Young females	LD_50_ and LD_90_, respectively	α-pinene, ∆^3^-carene and terpinen-4-ol	[43]
Apiaceae	*Deverra scoparia* Coss. & Durieu	Essential oil	-	D	larvae and adults	Mortalities of 98% (larvae) and 97% (adults)	-	[88]
Apiaceae	*Ferula gumosa Boiss.*	Essential oil	6.98 and 6.52 μL/L air	C,J	Eggs and adults, respectively	LD_50_	β-pinene (50.1%), α-pinene (14.9%), δ-3-carene (6.7%)	[86]
Apiaceae	*Foeniculum vulgare* Mill.	Seed essential oil vapors	5.75 µL/L (females), 1.17 µL/L (eggs)	J	Eggs and adults	LD_50_	-	[84]
Apiaceae	*Foeniculum vulgare* Mill.	Essential oil	1.17%	E	Adults	LD_50_	-	[83]
Apiaceae	*Heracleum persicum* Desf. Ex. Fisch.	Fruit essential oils vapors	3.15 µL/L (females)-1.53 µL/L (eggs)	J	Eggs and adults	LD_50_	-	[84]
Apiaceae	*Heracleum persicum* Desf. Ex. Fisch.	Essential oil	1.53%	E	Adults	LD_50_	-	[83]
Apiaceae	*Smyrnium olusatrum* L.	Inflorescence extract	1.9 and 42.7 µg/mL, respectively for isolated compounds	D	Adult females	LD_50_ (chronic toxicity after 5 days)	Isolation of isofuranodiene and germacrone, separately evaluated	[87]
Apocynaceae	*Vinca erecta* Regel & Schmalh	Aerial part extract	1%	G	Adult females	Mortality between 50 and 80%	-	[99]
Apocynaceae	*Vinca minor* L.	Aerial part extract	1%	G	Adult females	Mortality between 0 and 20%	-	[99]
Araceae	*Arum korolkovii* Regel	Aerial part extract	1%	G	Adult females	Mortality between 0 and 20%	-	[99]
Asclepiadaceae	*Calotropis gigantea* W.T. Aiton	Leaf extract	5000 ppm	G	Adults	Mortality between 61 and 80%	-	[71]
Asclepiadaceae	*Calotropis gigantea* W.T. Aiton	Leaf extract	2500 ppm	G	Adults	Mortality between 40 and 60%	-	[71]
Asteraceae	*Achillea mellifolium* L.	Essential oil from aerial part	1.208% *v*/*v* (leaf dipping) and 1.801 µL/L air (fumigation)	G,J	Adult females	LD_50_	Piperitone (12.8%), *p*-cymene (10.6%)	[64]
Asteraceae	*Achillea millefolium* L.	Aerial part extract	1%	G	Adult females	Mortality between 0 and 20%	-	[99]
Asteraceae	*Acroptilon repens* (L.) DC.	Aerial part extract	1%	G	Adult females	Mortality between 50 and 80%	-	[99]
Asteraceae	*Ajania fastigiata* (C. Winkler) Poljakov	Aerial part extract	1%	G	Adult females	Mortality between 0 and 20%	-	[99]
Asteraceae	*Anaphalis rosea-alba* Krasch.	Aerial part extract	1%	G	Adult females	Mortality between 20 and 50%	-	[99]
Asteraceae	*Anthemis nobilis* L.	Essential oil	19 × 10^−3^ µL/mL of air	J	Adults	69% of mortality	-	[42]
Asteraceae	*Anthemis vulgaris* L.	Flower extract	7–50%	A,B	Adult females	Mortalities of 92.37% and 92.34%, respectively	-	[60]
Asteraceae	*Anthemis vulgaris* L.	Leaf extract	13–50%	A,B	Adult females	Mortalities of 82.33% and 76.63%, respectively	-	[60]
Asteraceae	*Artemisia absinthium* L.	Essential oil	19 × 10^−3^ µL/mL of air	J	Adults	97% of mortality	-	[42]
Asteraceae	*Artemisia absinthium* L.	Essential oil	0.043 mg/cm^2^	A	-	LD_50_	-	[45]
Asteraceae	*Artemisia absinthium* L.	Aerial part extract	1%	G	Adult females	Mortality between 20 and 50%	-	[99]
Asteraceae	*Artemisia aschurbajewii* C. Winkl.	Aerial part extract	1%	G	Adult females	Mortality between 0 and 20%	-	[99]
Asteraceae	*Artemisia cinae* O. Berg & C.F. Schmidt ex. Pljakov	Leaf extract	1326.53 ppm	A,B	Adult females	LD_50_	-	[60]
Asteraceae	*Artemisia compacta* Fisch. Ex. Besser	Aerial part extract	1%	G	Adult females	Mortality between 20 and 50%	-	[99]
Asteraceae	*Artemisia dracunculus* L.	Aerial part extract	1%	G	Adult females	Mortality between 20 and 50%	-	[99]
Asteraceae	*Artemisia judaica* L.	Leaves (acetone extract)	0.56 μg/mL	C,G	Adult females	LD_50_	-	[39]
Asteraceae	*Artemisia panciflora*	Aerial part extract	1%	G	Adult females	Mortality between 50 and 80%	-	[99]
Asteraceae	*Artemisia vulgaris* L.	Leaf extract	15-50%	A,B	Adult females	Mortalities of 54.13% and 75.12%, respectively	-	[60]
Asteraceae	*Artemisia vulgaris* L.	Aerial part extract	1%	G	Adult females	Mortality between 50 and 80%	-	[99]
Asteraceae	*Calendula officinalis* L.	Aerial part extract	1%	G	Adult females	Mortality between 50 and 80%	-	[99]
Asteraceae	*Chamomilla recutita* L.	Essential oil	0.65–1.17%	C	Adults ggs	LD_50_	α-basabolol oxide (35.25%), Trans β-farersene (7.76%)	[89]
Asteraceae	*Chrisanthemum coronarium* L.	Essential oil	-	D	Larvae and adults	Mortalities of 88% (larvae) and 93% (adults)	-	[88]
Asteraceae	*Handelia trichopylla* Heimerl	Aerial part extract	1%	G	Adult females	Mortality between 0 and 20%.	-	[99]
Asteraceae	*Hertia cheirifolia* (L.) Kuntze	Essential oil	3.43 mg/L	E	Adult females	LD_50_ and side-effect over fecundity		[46]
Asteraceae	*Hertia cheirifolia* (L.) Kuntze	Essential oil	-	D	Larvae and adults	Mortalities of 81% (larvae) and 89% (adults)	-	[88]
Asteraceae	*Hieracium dschirgalanicum* E. Nikit.	Aerial part extract	1%	G	Adult females	Mortality between 0 and 20%	-	[99]
Asteraceae	*Inula helenium* L.	Aerial part extract	1%	G	Adult females	Mortality between 20 and 50%	-	[99]
Asteraceae	*Jurinea capussi* Franch.	Aerial part extract	1%	G	Adult females	Mortality between 0 and 20%	-	[99]
Asteraceae	*Lamyropappus schakaptaricus* Knorr & Tamamsch.	Aerial part extract	1%	G	Adult females	Mortality between 0 and 20%	-	[99]
Asteraceae	*Matricaria chamomilla* L.	Aerial part extract	1%	G	Adult females	Mortality between 0 and 20%	-	[99]
Asteraceae	*Matricaria matricarioides* (Less.) Porter	Aerial part extract	1%	G	Adult females	Mortality between 20 and 50%	-	[99]
Asteraceae	*Matricaria recutita* L.	Aerial part extract	1%	G	Adult females	Mortality between 0 and 20%	-	[99]
Asteraceae	*Pseudoglossanthis litwinowii* (Tzvel.) R. Kam.	Aerial part extract	1%	G	Adult females	Mortality between 0 and 20%	-	[99]
Asteraceae	*Pyrethrum alatavicum* O. & B. Fedtsch.	Aerial part extract	1%	G	Adult females	Mortality between 20 and 50%	-	[99]
Asteraceae	*Pyrethrum branchanthemoides* R. Kam. & Lazkov	Aerial part extract	1%	G	Adult females	Mortality between 20 and 50%	-	[99]
Asteraceae	*Pyrethrum cinerariifolium* Trev.	Aerial part extract	1%	G	Adult females	Mortality between 20 and 50%	-	[99]
Asteraceae	*Pyrethrum sovetkinae* Kovalevsk	Aerial part extract	1%	G	Adult females	Mortality between 50 and 80%	-	[99]
Asteraceae	*Pyrethrum sussamyrense* Lazkov	Root extract	1%	G	Adult females	Mortality between 0 and 20%	-	[99]
Asteraceae	*Santolina africana* Jord. & Fourr.	Essential oil	2.35 mg/L	E	Adult females	LD_50_ and side-effect over fecundity	Terpinen-4-ol (54.96%)	[46]
Asteraceae	*Santolina africana* Jord. & Fourr.	Essential oil	-	D	Larvae and adults	Mortalities of 77% (larvae) and 68% (adults)	-	[88]
Asteraceae	*Senecio saposhnikovii* Krasch et. Schipcz.	Aerial part extract	1%	G	Adult females	Mortality between 50 and 80%	-	[99]
Asteraceae	*Seriphidium herba-album* (Asso) Sojak	Essential oil	-	D	Larvae and adults	Mortalities of 54% (larvae) and 37% (adults)	-	[88]
Asteraceae	*Tagetes minuta* L.	Aerial part extract	1%	G	Adult females	Mortality between 50 and 80%	-	[99]
Asteraceae	*Tanacetopsis ferganensis* Kovalevsk	Aerial part extract	1%	G	Adult females	Mortality between 20 and 50%	-	[99]
Asteraceae	*Tanacetopsis setacea* Kovalevsk	Aerial part extract	1%	G	Adult females	Mortality between 0 and 20%	-	[99]
Asteraceae	*Tanacetopsis submarginata* Kovalevsk	Aerial part extract	1%	G	Adult females	Mortality between 0 and 20%	-	[99]
Asteraceae	*Tanacetum boreale* Fisch. Ex. DC.	Aerial part extract	1%	G	Adult females	Mortality between 20 and 50%	-	[99]
Asteraceae	*Tanacetum pseudoachillea* C. Winkl.	Aerial part extract	1%	G	Adult females	Mortality between 50 and 80%	-	[99]
Asteraceae	*Tanacetum vulgare* L.	Essential oil	4%	A	-	75.6% of mortality	-	[45]
Asteraceae	*Thitonia diversifolia* Hemsl.	Methanolic extract	150 µg/cm^3^	D	Adult females	Mortality less than 50%	-	[92]
Asteraceae	*Tripleurospermum inodorum* Sch. Bip.	Aerial part extract	1%	G	Adult females	Mortality between 0 and 20%	-	[99]
Asteraceae	*Xanthium strumarium* L.	Fruit extract	9-50%	A,B	Adult females	Mortalities of 68.24% and 85.88%, respectively	-	[60]
Asteraceae	*Xanthium strumarium* L.	Leaf extract	11-50%	A,B	Adult females	Mortalities of 52.48% and 79.85%, respectively	-	[60]
Berveridaceae	*Berberis iliensis* Popov	Aerial part extract	1%	G	Adult females	Mortality between 20 and 50%	-	[99]
Bignonianceae	*Jacaranda obtusifolia* Bonpl.	Leaf extract	0.06%	C,G	Adult females	Mortality of 64.4%		[124]
Boraginaceae	*Echium vulgare* L.	Aerial part extract	1%	G	Adult females	Mortality between 50 and 80%	-	[99]
Boraginaceae	*Onosma visianii* Clem.	Root extract	2.6 µg/mL	D	Adult females	LD_50_ (chronic toxicity after 5 days)	Shikonin derivatives (naphthoquinones), i.e., isobutylshikonin and isovalerylshikonin	[93]
Brassicaceae	*Armoracia rusticana* G. Gaertn., B. Mey. & Scherb.	Aerial part extract	1%	G	Adult females	Mortality between 50 and 80%	-	[99]
Brassicaceae	*Barbarea vulgaris* W.T. Aiton	Aerial part extract	1%	G	Adult females	Mortality between 20 and 50%	-	[99]
Brassicaceae	*Capsella bursa-pastoris* L.	Aerial part extract	1%	G	Adult females	Mortality between 0 and 20%	-	[99]
Brassicaceae	*Cardaria repens* Schrenk	Aerial part extract	1%	G	Adult females	Mortality between 0 and 20%	-	[99]
Brassicaceae	*Lepidium latifolium* L.	Aerial part extract	1%	G	Adult females	Mortality between 50 and 80%	-	[99]
Burseraceae	*Boswellia carterii* Birdw.	Essential oil	0.1%	G	Adult females	24.8% of mortality	-	[112]
Burseraceae	*Commiphora myrrha* (Nees) Engl.	Essential oil	0.1%	G	Adult females	22.8% of mortality	-	[112]
Burseraceae	*Protium bahianum* Daly	Fresh and old resin essential oils	-	J	Adult females	Mortalities of 79.6% (fresh resin) and 59% (old resin)	-	[97]
Caesalpiniaceae	*Cassia mimosoides* L.	Leaf extract	5000 ppm	G	Adults	More than 80% of mortality	-	[71]
Caesalpiniaceae	*Cassia mimosoides* L.	Leaf extract	2500 ppm	G	Adults	Mortality between 61 and 80%	-	[71]
Caesalpiniaceae	*Cassia occidentalis* L.	Whole plant extract	5000 ppm	G	Adults	Mortality between 61 and 80%	-	[71]
Caesalpiniaceae	*Cassia occidentalis* L.	Whole plant extract	2500 ppm	G	Adults	Mortality between 61 and 80%	-	[71]
Caesalpiniaceae	*Cassia tora* L.	Whole plant extract	5000 ppm	G	Adults	More than 80% of mortality	-	[71]
Caesalpiniaceae	*Cassia tora* L.	Whole plant extract	2500 ppm	G	Adults	Mortality between 61 and 80%	-	[71]
Campanulaceae	*Codonopsis clematidea* Schrenk	Aerial part extract	1%	G	Adult females	Mortality between 50 and 80%	-	[99]
Cannabaceae	*Cannabis sativa L.*	Essential oil from panicles	0.10%	G	Adult females	83.28% of mortality	β-myrcene (18.5%), trans-caryophyllene (35.6%)	[98]
Cannabaceae	*Cannabis sativa* L.	Aerial part and root extracts	1%	G	Adult females	Mortality between 50 and 80%	-	[99]
Cannabaceae	*Humulus lupulus* L.	Flower extract	5-50%	A,B	Adult females	Mortalities of 56.37% and 67.84%, respectively	-	[60]
Cappandaceae	*Clome viscosa* L.	Whole plant extract	5000 ppm	G	Adults	More than 80% of mortality	-	[71]
Cappandaceae	*Clome viscosa* L.	Whole plant extract	2500 ppm	G	Adults	Mortality between 61 and 80%	-	[71]
Capparidaceae	*Boscia senagalensis* (Pers.) Lam. Ex. Poir.	Leaf extract	5000 ppm	G	Adults	More than 80% of mortality	-	[71]
Capparidaceae	*Boscia senagalensis* (Pers.) Lam. Ex. Poir.	Leaf extract	2500 ppm	G	Adults	Mortality between 61 and 80%	-	[71]
Caprifoliaceae	*Sambucus nigra* L.	Aerial part extract	1%	G	Adult females	Mortality between 0 and 20%	-	[99]
Caryophyllaceae	*Saponaria officinalis* L.	Root extract	0.31% (eggs), 1.18% (adulst), 0.91% (oviposition) w/v	I	Eggs, adults and oviposition	LD_50_	-	[66]
Caryophyllaceae	*Silene sussamyrica* Lazkov	Aerial part extract	1%	G	Adult females	Mortality between 0 and 20%	-	[99]
Chenopodiaceae	*Anabasis aphylla* L.	Seed and bark extracts	1%	G	Adult females	Mortality between 50 and 80%	-	[99]
Chenopodiaceae	*Anthochlamis tianschanica* Iljin	Aerial part extract	1%	G	Adult females	Mortality between 20 and 50%	-	[99]
Chenopodiaceae	*Chenopodium álbum* (L.) Mosc. Ex. Moq.	Flower and leaf extracts	8–50%.	A,B	Adult females	Mortalities of 96.99% and 91.15%, respectively	-	[60]
Combretaceae	*Cobretum micranthum* G. Don	Whole plant extract	5000 ppm	G	Adults	More than 80% of mortality	-	[71]
Combretaceae	*Cobretum micranthum* G. Don	Whole plant extract	2500 ppm	G	Adults	More than 80% of mortality	-	[71]
Combretaceae	*Combretum glutinosum* Perr. Ex. DC.	Leaf extract	5000 ppm	G	Adults	More than 80% of mortality	-	[71]
Combretaceae	*Combretum glutinosum* Perr. Ex. DC.	Leaf extract	2500 ppm	G	Adults	More than 80% of mortality	-	[71]
Combretaceae	*Combretum glutinosum* Perr. Ex. DC.	Stem extract	5000 ppm	G	Adults	Mortality between 61 and 80%	-	[71]
Combretaceae	*Combretum glutinosum* Perr. Ex. DC.	Stem extract	2500 ppm	G	Adults	Mortality between 40 and 60%	-	[71]
Combretaceae	*Guiera senegalensis* J.F. Gmel.	Leaf extract	5000 ppm	G	Adults	More than 80% of mortality	-	[71]
Combretaceae	*Guiera senegalensis* J.F. Gmel.	Leaf extract	2500 ppm	G	Adults	Mortality between 61 and 80%	-	[71]
Combretaceae	*Guiera senegalensis* J.F. Gmel.	Stem extract	5000 ppm	G	Adults	Mortality between 61 and 80%	-	[71]
Combretaceae	*Guiera senegalensis* J.F. Gmel.	Stem extract	2500 ppm	G	Adults	Mortality between 40 and 60%	-	[71]
Combretaceae	*Piloitigma vetilicolin*	Whole plant extract	5000 ppm	G	Adults	More than 80% of mortality	-	[71]
Combretaceae	*Piloitigma vetilicolin*	Whole plant extract	2500 ppm	G	Adults	Mortality between 61 and 80%	-	[71]
Convolvulaceae	*Convolvulus arvensis* L.	Aerial part extract	1%	G	Adult females	Mortality between 20 and 50%	-	[99]
Convolvulaceae	*Convolvulus krauseanus* Regel. & Schmalh	Root extract	1%	G	Adult females	Mortality between 80 and 100%	-	[99]
Convolvulaceae	*Ipomaea asarifolia* (Desr.) Roem. & Schult.	Whole plant extract	5000 ppm	G	Adults	More than 80% of mortality	-	[71]
Convolvulaceae	*Ipomaea asarifolia* (Desr.) Roem. & Schult.	Whole plant extract	2500 ppm	G	Adults	Mortality between 40 and 60%	-	[71]
Convolvulaceae	*Ipomaea sp.* L.	Whole plant extract	5000 ppm	G	Adults	More than 80% of mortality	-	[71]
Convolvulaceae	*Ipomaea sp.* L.	Whole plant extract	2500 ppm	G	Adults	Mortality between 61 and 80%	-	[71]
Cupressaceae	*Cupressus macrocarpa* Hartw. ex Gordon	Leaf extract	5.69 µL/L air	A	Adult females	LD_50_	β-citronellol (35.92%)	[55]
Cupressaceae	*Cupressus sempervirens* L.	Essential oil	0.1%.	G	Adult females	28.9% of mortality	-	[112]
Cupressaceae	*Juniperus communis* L.	Essential oil	0.1%.	G	Adult females	42.6% of mortality	-	[112]
Cupressaceae	*Juniperus phoenicea* L.	Essential oil	-	D	Larvae and adults	Mortalities of 60% (larvae) and 56% (adults)	-	[88]
Cupressaceae	*Thuja orientalis* L.	Leaf extract	7.51 µL/L air	A	Adult females	LD_50_	α-pinene (35.49%)	[55]
Elaeagnaceae	*Elaeagnus angustifolia* L.	Aerial part extract	1%	G	Adult females	Mortality between 20 and 50%	-	[99]
Equisetaceae	*Equisetum arvense* L.	Aerial part extract	1%	G	Adult females	Mortality between 0 and 20%	-	[99]
Euforbiaceae	*Jatropha curcas* L.	Leaf extract	0.06%	C,G	Adult females	Mortality of 63.3%	-	[124]
Euphorbiaceae	*Chrozophora oblongifolia (Delile) Spreng.*	Whole plant extract	312.72 and 206.91 ppm	G	Adult females and larvae	LD_50_	7-O-β-D-[2”,6”-bis(4-hydroxy-E-cinnamoyl)] glucopyranoside, apigenin 7-O-ß-D-glucopyranoside isolated from butanol fration	[125]
Euphorbiaceae	*Cnidoscolus aconitifolius* (Mill) I.M. Johnst.	Leaf extract	2000 µg/mL	C,G	Adult females	92% of mortality	-	[61]
Euphorbiaceae	*Euphorbia ferganensis* B. Fedtsch.	Root extract	1%	G	Adult females	Mortality between 0 and 20%	-	[99]
Euphorbiaceae	*Euphorbia kansui* S.L. Liou S.B. Ho	Root extract	3-5 g/L	C	Adult females	Mortalities of 27% and 55%, respectively	3-O-(2,3-dimethylbutanoyl)-13-dodecanoylingenol y 3-O-(2′E,4′Z-decadienoyl)-ingenol	[100]
Fabaceae	*Acacia cyanophylla* Lindl.	Essential oil	-	D	Larvae and adults	Mortalities of 58% (larvae) and 26% (adults)	-	[88]
Fabaceae	*Amnopiptanthus nanus* (M. pop) Cheng	Pod extract	1%	G	Adult females	Mortality between 50 and 80%	-	[99]
Fabaceae	*Bowdichia virgilioides* Kunth	Leaf extract	0.06% w/v	C,G	Adult females	Mortality of 64.4%		[126]
Fabaceae	*Gleditschia* spp.	Aerial part extract	1%	G	Adult females	Mortality between 0 and 20%	-	[99]
Fabaceae	*Glycirrhisa uralensis* L.	Aerial part extract	1%	G	Adult females	Mortality between 0 and 20%	-	[99]
Fabaceae	*Hedysarum cephalotes* Franchet	Whole plant extract	1%	G	Adult females	Mortality between 20 and 50%	-	[99]
Fabaceae	*Hedysarum daraut-kurganicum* Sultanova	Aerial part extract	1%	G	Adult females	Mortality between 50 and 80%	-	[99]
Fabaceae	*Hymenaea courbaril* L.	Leaf extract	0.06% w/v	C,G	Adult females	Mortality of 59.4%		[126]
Fabaceae	*Medicago minima* L.	Aerial part extract	1%	G	Adult females	Mortality between 50 and 80%	-	[99]
Fabaceae	*Melilotus officinalis* L.	Aerial part extract	1%	G	Adult females	Mortality between 0 and 20%	-	[99]
Fabaceae	*Millettia pinnata L.*	Laef oil	0.004%	C	Adult females	LD_50_ (after 4 days)	-	[101]
Fabaceae	*Oxytropis rosea* Bunge	Aerial part extract	1%	G	Adult females	Mortality between 20 and 50%	-	[99]
Fabaceae	*Sophora korolkovii* Koehne.	Aerial part extract	1%	G	Adult females	Mortality between 0 and 20%	-	[99]
Fabaceae	*Sophora secundiflora* (Ortega) Lag. Ex. DC.	Essential oil	-	D	Larvae and adults	Mortalities of 68% (larvae) and 61% (adults)	-	[88]
Fabaceae	*Vicia cracca* L.	Aerial part extract	1%	G	Adult females	Mortality between 0 and 20%	-	[99]
Geraniaceae	*Pelargonium graveolens* L’Her	Leaf extract	12.27 µL/L air	A	Adult females	LD_50_	terpinen-4-ol (20.29%)	[55]
Geraniaceae	*Pelargonium graveolens* L’Hér.	Essential oil	19 × 10^−3^ µL/mL of air.	K	Adults	100% of mortality	-	[42]
Geraniaceae	*Pelargonium graveolens* L’Hér.	Essential oil	-	D	Larvae and adults	Mortalities of 78% (larvae) and 70% (adults)	-	[88]
Geraniaceae	*Pelargonium roseum* Willd	Essential oil	0.1%.	G	Adult females	30% of mortality	-	[112]
Gramineae	*Chrysopogon zizanioides (L.)*	Essential oil	18.82 μg/mL	J	Adult females	LD_50_	-	[127]
Gramineae	*Cymbopogon citratus* (DC.) Stapf	Essential oil	19 × 10^−3^ µL/mL of air.	J	Adults	100% of mortality	-	[42]
Gramineae	*Cymbopogon citratus* (DC.) Stapf	Essential oil	0.1%.	G	Adult females	17.8% of mortality	-	[112]
Gramineae	*Cymbopogon flexuosus (Nees ex Steud.) W. Watson*	Essential oil	17.23 μg/mL	J	Adult females	LD_50_	-	[127]
Gramineae	*Cymbopogon Martini* (Roxb.) W. Watson	Essential oil	19 × 10^−3^ µL/mL of air	J	Adults	67% of mortality	-	[42]
Gramineae	*Cymbopogon nardus* (L) Rendle	Essential oil	19 × 10^−3^ µL/mL of air	J	Adults	99% of mortality	-	[42]
Gramineae	*Cymbopogon nardus* (L) Rendle	Essential oil	22.5 µg/cm^3^	C,J	Adults	LD_50_	-	[68]
Gramineae	*Cymbopogon winterianus* Jowitt ex. Bor	Essential oil	0.1%	G	Adult females	27.6% of mortality	-	[112]
Gramineae	*Lolium perenne* L.	Leaf and flower methanolic extracts	6-50%	A,B	Adult females	Mortalities of 91.43% and 93.5%, respectively	-	[60]
Guttiferae	*Hypericum perforatum* L.	Aerial part extract	1%	G	Adult females	Mortality between 50 and 80%	-	[99]
Iridaceae	*Iris sogdiana* Regel.	Leaf extract	1%	G	Adult females	Mortality between 0 and 20%	-	[99]
Juglandecaea	*Juglans regia* L.	Leaf extract	12% v/w	C,G	Adult females and nymphs	Mortality between 83 and 90%	-	[128]
Lamiaceae	*Acinos thymoides* (L.) Moench	Aerial part extract	1%	G	Adult females	Mortality between 50 and 80%	-	[99]
Lamiaceae	*Ajuga australis* R.Br.	Leaf extract	1%	C	-	Mortality between 20 and 49%	-	[103]
Lamiaceae	*Callicarpa pedunculata* R.Br.	Leaf extract	1%	C	-	Mortality between 20 and 49%	-	[103]
Lamiaceae	*Ceratanthus longicornis* (F.Muell.) G. Taylor	Leaf extract	1%	C	-	100% of mortality	-	[103]
Lamiaceae	*Clerodendrum floribundum* R.Br.	Leaf extract	1%	C	-	Mortality between 50 and 89%	-	[103]
Lamiaceae	*Clerodendrum inerme* (L.) Gaertn.	Leaf extract	1%	C	-	Mortality between 50 and 89%	-	[103]
Lamiaceae	*Clerodendrum tomentosum* (Vent.) R.Br.	Leaf extract	1%	C	-	Mortality between 50 and 89%	-	[103]
Lamiaceae	*Clerodendrum traceyi* F. Muell.	Leaf extract	1%	C	-	100% of mortality	-	[103]
Lamiaceae	*Faradaya albertissii* F. Muell.	Leaf extract	1%	C	-	Less than 20% of mortality	-	[103]
Lamiaceae	*Faradaya splendida* F. Muell.	Leaf extract	1%	C	-	Mortality between 50 and 89%	-	[103]
Lamiaceae	*Glossocarya calcicola* Domin.	Leaf extract	1%	C	-	Less than 20% of mortality	-	[103]
Lamiaceae	*Glossocarya hemiderma* Benth.	Leaf extract	1%	C	-	Mortality between 20 and 49%	-	[103]
Lamiaceae	*Gmelina leichardtii* (F.Muell.) Benth	Leaf extract	1%	C	-	Mortality between 90 and 99%	-	[103]
Lamiaceae	*Hemiandra australis* B.J. Conn.	Leaf extract	1%	C	-	Mortality between 20 and 49%	-	[103]
Lamiaceae	*Hemiandra leiantha* Benth.	Leaf extract	1%	C	-	Less than 20% of mortality	-	[103]
Lamiaceae	*Hemiandra pungens* R.Br.	Leaf extract	1%	C	-	Mortality between 20 and 49%	-	[103]
Lamiaceae	*Hemigenia humilis* Benth.	Leaf extract	1%	C	-	Mortality between 20 and 49%	-	[103]
Lamiaceae	*Hemigenia sericea* Benth.	Leaf extract	1%	C	-	Less than 20% of mortality	-	[103]
Lamiaceae	*Hemigenia westringioides* Benth.	Leaf extract	1%	C	-	Less than 20% of mortality	-	[103]
Lamiaceae	*Hyssopus officinalis* L.	Aerial part extract	1%	G	Adult females	Less than 20% of mortality	-	[99]
Lamiaceae	*Hyssopus officinalis* L.	Essential oil	0.1%.	G	Adult females	28.1% of mortality	-	[112]
Lamiaceae	*Lachnostachys eriobotrya* (F. Muell.) Druce	Leaf extract	1%	C	-	Less than 20% of mortality	-	[103]
Lamiaceae	*Lavandula angustifolia* Mill.	Leaf extract	4.93 µL/L	J	Adult females	LD_50_	1,8-cineole, camphor, β-pinene	[129]
Lamiaceae	*Lavandula latifolia* Medik.	Essential oil from twigs with leaves andflowers	0.20–0.25% *v*/*v*	A,C	Adult females	Mortality between 95 and 100%	linalool (37.8%), 1,8-cineole (24.9%), camphor (18.7%)	[56]
Lamiaceae	*Lavandula officinalis* Chaix	Essential oil	19 × 10^−3^ µL/mL of air	J	Adults	97% of mortality	-	[42]
Lamiaceae	*Lavandula officinalis* Chaix	Essential oil	-	D	Larvae and adults	Mortalities of 38% (larvae) and 41% (adults)	-	[88]
Lamiaceae	*Lavandula vera* DC.	Essential oil	0.1%	G	Adult females	26.1% of mortality	-	[112]
Lamiaceae	*Leonorus turkestanicus* V. Krecz. & Kupr.	Aerial part extract	1%	G	Adult females	Mortality between 50 and 80%	-	[99]
Lamiaceae	*Lycopus australis* R. Br.	Leaf extract	1%	C	-	Mortality between 20 and 49%	-	[103]
Lamiaceae	*Majorana hortensis* Moench	Essential oil	1.84–6.26%	C	Adults and eggs (respectively)	LD_50_	terpinen-4-ol (23.86%), p-cymene (23.40%) and sabinene (10.90%)	[89]
Lamiaceae	*Melissa officinalis* L.	Aerial part extract	1%	G	Adult females	Mortality between 0 and 20%	-	[99]
Lamiaceae	*Menta spicata L.*	Essential oil	15 μL/L	C,J	Eggs	LD_50_	carvone (68.5%)	[130]
Lamiaceae	*Mentha arvensis* L.	Aerial part extract	1%	G	Adult females	Mortality between 80 and 100%	-	[99]
Lamiaceae	*Mentha longifolia L.*	Essential oil	11.08 μg/mL	J	Adult females	LD_50_	-	[127]
Lamiaceae	*Mentha piperita* L.	Essential oil	19 × 10^−3^ µL/mL of air	J	Adults	100% of mortality	-	[42]
Lamiaceae	*Mentha piperita* L.	Essential oil	22.8 µg/cm^3^	C,J	Adults	LD_50_	-	[68]
Lamiaceae	*Mentha piperita* L.	Essential oil	0.1%.	G	Adult females	23.7% of mortality	-	[112]
Lamiaceae	*Mentha piperita L.*	Essential oil	15.86 μg/mL	J	Adult females	LD_50_	-	[127]
Lamiaceae	*Mentha pulegium* L.	Essential oil	19 × 10^−3^ µL/mL of air	J	Adults	100% of mortality	-	[42]
Lamiaceae	*Mentha pulegium* L.	Essential oil	23.7 µg/cm^3^	J	Adults	LD_50_	-	[68]
Lamiaceae	*Mentha pulegium* L.	Essential oil	-	D	Larvae and adults	Mortalities of 90% (larvae) and 91% (adults)	-	[88]
Lamiaceae	*Mentha spicata* L.	Essential oil	19 × 10^−3^ µL/mL of air	J	Adults	100% of mortality	-	[42]
Lamiaceae	*Mentha spicata* L.	Essential oil	38.8 µg/cm^3^	C,J	Adults	LD_50_	-	[68]
Lamiaceae	*Mentha spicata* L.	essential oil from leaves	7.53 µL/L air	C,J	Adult females	LD_50_	carvone (59.4%), limonene (9.8%), 1,8-cineole (7.4%)	[85]
Lamiaceae	*Mentha sylvestris* L.	Aerial part extract	1%	G	Adult females	Mortality between 20 and 50%	-	[99]
Lamiaceae	*Microcorys capitata* (Bartl.) Benth.	Leaf extract	1%	C	-	Less than 20% of mortality	-	[103]
Lamiaceae	*Microcorys* sp.	Leaf extract	1%	C	-	Less than 20% of mortality	-	[103]
Lamiaceae	*Micromeria fruticosa* L.	Essential oil vapors	2 µL/L of air	J	Adults and nimphs	96.7% of mortality	-	[105]
Lamiaceae	*Micromeria fruticosa* L.	Essential oil vapors	2 µL/L	J	Adults and nimphs	96.7% of mortality	-	[105]
Lamiaceae	*Nepeta racemosa* L.	Essential oil vapors	2 uL/L of air	J	Adults and nimphs	95% of mortality	-	[105]
Lamiaceae	*Nepeta racemosa* L.	Essential oil vapors	2 µL/L	J	Adults and nimphs	95% of mortality	-	[105]
Lamiaceae	*Ocimum basilicum* L.	Essential oil	19 × 10^−3^ µL/mL of air	J	Adults	88% of mortality	-	[42]
Lamiaceae	*Ocimum basilicum* L.	Essential oil	39.5 µg/cm^3^	C,J	Adults	LD_50_	-	[68]
Lamiaceae	*Ocimum basilicum* L.	Essential oil	0.1%	G	Adult females	21% of mortality	-	[112]
Lamiaceae	*Ocimum basilicum* L.	Essential oil	0.6 μL/L	C,J	Adult females	LD_50_	linalool (65.7%)	[130]
Lamiaceae	*Origanum majorana* L.	Essential oil	19 × 10^−3^ µL/mL of air	J	Adults	92% of mortality	-	[42]
Lamiaceae	*Origanum majorana* L.	Essential oil	0.1%	G	Adult females	7.1% of mortality	-	[112]
Lamiaceae	*Origanum vulgare* L.	Essential oil	8.52 µL/L air	A	Adult females	LD_50_	pulegone (77.45%)	[55]
Lamiaceae	*Origanum vulgare* L.	Aerial part extract	1%	G	Adult females	Mortality between 20 and 50%	-	[99]
Lamiaceae	*Origanum vulgare* L.	Essential oil vapors	2 uL/L of air	J	Adults and nimphs	95% of mortality	-	[105]
Lamiaceae	*Origanum vulgare* L.	Essential oil vapors	2 µL/L	J	Adults and nimphs	95% of mortality	-	[105]
Lamiaceae	*Otostelgia olgae* (Regel.) Korsch.	Aerial part extract	1%	G	Adult females	Mortality between 0 and 20%	-	[99]
Lamiaceae	*Pityrodia bartlingii* (Lehm.) Benth.	Leaf extract	1%	C	-	Mortality between 50 and 89%	-	[103]
Lamiaceae	*Pityrodia verbascina* (F. Muell.) Benth.	Leaf extract	1%	C	-	Less than 20% of mortality	-	[103]
Lamiaceae	*Plectranthus actites* P.I. Forst.	Leaf extract	1%	C	-	Mortality between 50 and 89%	-	[103]
Lamiaceae	*Plectranthus alloplectus* S.T. Blake	Leaf extract	1%	C	-	Mortality between 20 and 49%	-	[103]
Lamiaceae	*Plectranthus amoenus* P.I. Forst	Leaf extract	1%	C	-	Less than 20% of mortality	-	[103]
Lamiaceae	*Plectranthus apreptus* S.T. Blake	Leaf extract	1%	C	-	Less than 20% of mortality	-	[103]
Lamiaceae	*Plectranthus argentatus* S.T. Blake	Leaf extract	1%	C	-	Less than 20% of mortality	-	[103]
Lamiaceae	*Plectranthus cremnus* B. J. Conn.	Leaf extract	1%	C	-	Mortality between 50 and 89%	-	[103]
Lamiaceae	*Plectranthus diversus* S.T. Blake	Leaf extract	1%	C	-	Mortality between 90 and 99%	-	[103]
Lamiaceae	*Plectranthus fasciculatus* P.I. Forst.	Leaf extract	1%	C	-	Less than 20% of mortality	-	[103]
Lamiaceae	*Plectranthus foetidus* Benth.	Leaf extract	1%	C	-	Less than 20% of mortality	-	[103]
Lamiaceae	*Plectranthus glabriflorus* P.I. Forst	Leaf extract	1%	C	-	Mortality between 90 and 99%	-	[103]
Lamiaceae	*Plectranthus gratus* S.T. Blake	Leaf extract	1%	C	-	Less than 20% of mortality	-	[103]
Lamiaceae	*Plectranthus graveolens* R.Br.	Leaf extract	1%	C	-	Mortality between 50 and 89%	-	[103]
Lamiaceae	*Plectranthus habrophyllus* P.I. Forst	Leaf extract	1%	C	-	100% of mortality	-	[103]
Lamiaceae	*Plectranthus* Koonyum Range	Leaf extract	1%	C	-	Mortality between 50 and 89%	-	[103]
Lamiaceae	*Plectranthus leiperi* P.I. Forst.	Leaf extract	1%	C	-	Mortality between 50 and 89%	-	[103]
Lamiaceae	*Plectranthus mirus* S.T. Blake	Leaf extract	1%	C	-	Less than 20% of mortality	-	[103]
Lamiaceae	*Plectranthus nitidus* P.I. Forst.	Leaf extract	1%	C	-	Mortality between 50 and 89%	-	[103]
Lamiaceae	*Plectranthus omissus* P. I. Forst.	Leaf extract	1%	C	-	Mortality between 50 and 89%	-	[103]
Lamiaceae	*Plectranthus parviflorus* Willd	Leaf extract	1%	C	-	Mortality between 50 and 89%	-	[103]
Lamiaceae	*Plectranthus scutellarioides* (L.) R.Br.	Leaf extract	1%	C	-	Mortality between 50 and 89%	-	[103]
Lamiaceae	*Plectranthus sp. buchanans* Fort	Leaf extract	1%	C	-	Less than 20% of mortality	-	[103]
Lamiaceae	*Plectranthus sp.* Hann Tableland	Leaf extract	1%	C	-	100% of mortality	-	[103]
Lamiaceae	*Plectranthus sp.* Pinnacle	Leaf extract	1%	C	-	Less than 20% of mortality	-	[103]
Lamiaceae	*Plectranthus spectabilis* S.T. Blake	Leaf extract	1%	C	-	Less than 20% of mortality	-	[103]
Lamiaceae	*Plectranthus suaveolens* S.T. Blake	Leaf extract	1%	C	-	Mortality between 90 and 99%	-	[103]
Lamiaceae	*Pogostemon cablin* Benth.	Essential oil	0.1%	G	Adult females	20.3% of mortality	-	[112]
Lamiaceae	*Premna acuminata* R.Br.	Leaf extract	1%	C	-	Mortality between 90 and 99%	-	[103]
Lamiaceae	*Premna serratifolia* L.	Leaf extract	1%	C	-	100% of mortality	-	[103]
Lamiaceae	*Prostanthera incisa* Benth.	Leaf extract	1%	C	-	Less than 20% of mortality	-	[103]
Lamiaceae	*Prostanthera lasianthos* Labill.	Leaf extract	1%	C	-	Mortality between 20 and 49%	-	[103]
Lamiaceae	*Prostanthera nivea* A. Cunn. Ex. Benth.	Leaf extract	1%	C	-	Less than 20% of mortality	-	[103]
Lamiaceae	*Prostanthera rotundifolia* R.Br.	Leaf extract	1%	C	-	Less than 20% of mortality	-	[103]
Lamiaceae	*Prostanthera spinosa* F. Muell.	Leaf extract	1%	C	-	Less than 20% of mortality	-	[103]
Lamiaceae	*Prostanthera stricta* R.T. Baker	Leaf extract	1%	C	-	Less than 20% of mortality	-	[103]
Lamiaceae	*Rosmarinus officinalis* L.	Essential oil	0.10, 0.15, 0.20, and 0.25%.	A,C	Adult females and eggs	Mortalities of 15%, 79%, 100% and 100% for females, respectively	1,8-cineole (26.7%), camphor (17.5%), α-pinene (18.6%), camphene (11.8%), myrcene (9%), bornyl acetate (4%), β-pinene (2.8%), humulene (0.5%), borneol (1.8%), β-caryophyllene (1.5%), linalool (1%), Verbennone (0.9%), α-terpineol (0.8%)	[53]
Lamiaceae	*Rosmarinus officinalis* L.	Essential oil	-	D	Larvae and adults	Mortalities of 61% (larvae) and 53% (adults)	-	[88]
Lamiaceae	*Rosmarinus officinalis* L.	Essential oil	10 mL/L	D	-	LD_50_	1,8-cineole and α-pinene (mortalities of 88 ± 4.8% and 32 ± 4.8%, respectively with each compound)	[104]
Lamiaceae	*Rosmarinus officinalis* L.	Essential oil	0.1%	G	Adult females	11.7% of mortality	-	[112]
Lamiaceae	*Salvia desertorum*	Aerial part extract	1%	G	Adult females	Mortality between 0 and 20%	-	[99]
Lamiaceae	*Salvia fruticosa* Mill.	Leaf extract	3.77 µL/L	J	Adult females	LD_50_		[129]
Lamiaceae	*Salvia officinalis* L.	Essential oil	19 × 10^−3^ µL/mL of air	J	Adults	100% of mortality	-	[42]
Lamiaceae	*Salvia officinalis* L.	Essential oil	0.10%. 0.15%. 0.20% and 0.25%	A,C	Adult females and eggs	100% of female mortality in all concentrations	α-tujone (42.5), 1,8-cineole (10.3%), camphor (11%), α-pinene (6.7%), camphene (6.5%), β-tujone (6.6%), myrcene (1.4%), bornyl acetate (0.7%), β-pinene (3.4%), humulene (2.4%), viridiflorol (2.2%), borneol (2%), β-caryophyllene (1.5%), cymene (1%)	[53]
Lamiaceae	*Salvia officinalis* L.	Essential oil	63.7 µg/cm^3^	C,J	Adults	LD_50_	-	[68]
Lamiaceae	*Salvia officinalis* L.	Essential oil	-	D	Larvae and adults	Mortalities of 61% (larvae) and 57% (adults)	-	[88]
Lamiaceae	*Salvia sclarea* L.	Essential oil	19 × 10^−3^ µL/mL of air	J	Adults	61% of mortality	-	[42]
Lamiaceae	*Salvia sclarea* L.	Aerial part extract	1%	G	Adult females	Mortality between 0 and 20%	-	[99]
Lamiaceae	*Salvia sclarea* L.	Essential oil	0.1%.	G	Adult females	71% of mortality	-	[112]
Lamiaceae	*Salvia vvedenskyi* E. Nikit.	Root extract	1%	G	Adult females	Mortality between 50 and 80%	-	[99]
Lamiaceae	*Satureja sahendica* Bornm.	Essential oil vapors	0.98 µL/L (females), 0.54 µL/L (eggs)	J	Eggs and adults	LD_50_	-	[84]
Lamiaceae	*Satureja sahendica* Bornm.	Essential oil	0.54%	E	Adults	LD_50_	-	[83]
Lamiaceae	*Scutellaria mollis* R. Br.	Leaf extract	1%	C	-	Mortality between 50 and 89%	-	[103]
Lamiaceae	*Stachys tschatkalensis* Knorr.	Aerial part extract	1%	G	Adult females	Mortality between 50 and 80%	-	[99]
Lamiaceae	*Teucrium racemosum* R. Br.	Leaf extract	1%	C	-	Mortality between 20 and 49%	-	[103]
Lamiaceae	*Thymbra capitata* (L.) Cav.	Essential oil	-	D	Larvae and adults	Mortalities of 61% (larvae) and 52% (adults)	-	[88]
Lamiaceae	*Thymus vulgaris* L.	Essential oil	19 × 10^−3^ µL/mL of air	J	Adults	93% of mortality	-	[42]
Lamiaceae	*Thymus vulgaris* L.	Essential oil	22.7 µg/cm^3^	C,J	Adults	LD_50_	-	[68]
Lamiaceae	*Thymus vulgaris* L.	Essential oil	0.1%	G	Adult females	62.2% of mortality	-	[112]
Lamiaceae	*Vitex lignum-vitae* Schauer	Leaf extract	1%	C	-	Mortality between 50 and 89%	-	[103]
Lamiaceae	*Viticipremna queenslandica* Munir	Leaf extract	1%	C	-	Mortality between 90 and 99%	-	[103]
Lamiaceae	*Westringia eremicola* A. Cunn. Ex. Benth.	Leaf extract	1%	C	-	Less than 20% of mortality	-	[103]
Lamiaceae	*Westringia glabra* R.Br.	Leaf extract	1%	C	-	Less than 20% of mortality	-	[103]
Lamiaceae	*Westringia saxatilis* B.J. Conn	Leaf extract	1%	C	-	Less than 20% of mortality	-	[103]
Lamiaceae	*Westringia viminalis* B.J. Conn & Tozer	Leaf extract	1%	C	-	Less than 20% of mortality	-	[103]
Lamiaceae	*Ziziphora clinopodioides* Lam.	Aerial part extract	1%	G	Adult females	Mortality between 0 and 20%	-	[99]
Lauraceae	*Cinnamomum zeylandicum* Blume	Essential oil	0.1%	G	Adult females	23.6% of mortality	-	[112]
Lauraceae	*Laurus nobilis* L.	Leaf extract	17–50%	A,B	Adult females	Mortalities of 66.11% and 69.72%, respectively	-	[60]
Lauraceae	*Laurus nobilis* L.	Essential oil	-	D	Larvae and adults	Mortalities of 63% (larvae) and 46% (adults)	-	[88]
Lauraceae	*Licaria puchury-major (Mart.) Kosterm.*	Essential oil	30.8 µg/mL	J	Adult females	LD_50_	safrole (38.8%), 1,8-cineole (21.7%)	[131]
Lilliaceae	*Convallaria majalis* L.	Root extract	1%	G	Adult females	Mortality between 0 and 20%	-	[99]
Malvaceae	*Abutilon theophasti* Medic.	Aerial part extract	1%	G	Adult females	Mortality between 50 and 80%	-	[99]
Malvaceae	*Corchorus sp.*	Whole plant extract	5000 ppm	G	Adults	Mortality between 61 and 80%	-	[71]
Malvaceae	*Corchorus sp.*	Whole plant extract	2500 ppm	G	Adults	Mortality between 61 and 80%	-	[71]
Malvaceae	*Hybiscus sp.*	Whole plant extract	5000 ppm	G	Adults	More than 80% of mortality	-	[71]
Malvaceae	*Hybiscus sp.*	Whole plant extract	2500 ppm	G	Adults	-	-	[71]
Malvaceae	*Malva pusilla* Sm.	Whole plant extract	1%	G	Adult females	Mortality between 50 and 80%	-	[99]
Meliaceae	*Azadirachta indica* A. Juss.	Commercialformulation	1%.	C,D	Adult females	97.5% of mortality	-	[37]
Meliaceae	*Azadirachta indica* A. Juss.	Leaf extract	5000 ppm	G	Adults	Mortality between 61 and 80%	-	[71]
Meliaceae	*Azadirachta indica* A. Juss.	Leaf extract	2500 ppm	G	Adults	Mortality between 61 and 80%	-	[71]
Meliaceae	*Melia azedarach* L.	Fruit extract	14–50%	A,B	Adult females	Mortalities of 74.57% and 76.45%, respectively	-	[60]
Meliaceae	*Melia azedarach* L.	Essential oil	-	D	Larvae and adults	Mortalities of 77% (larvae) and 75% (adults)	-	[88]
Meliaceae	*Melia azedarach* L.	Acetone and petroleum ether methanolic extracts	-	C	Larvae	Lethal and fecundity effects	-	[107]
Mimosaceae	*Prosopis chinensis* (Molina) Stuntz	Leaf extract	5000 ppm	G	Adults	More than 80% of mortality	-	[71]
Mimosaceae	*Prosopis chinensis* (Molina) Stuntz	Leaf extract	2500 ppm	G	Adults	Mortality between 61 and 80%	-	[71]
Mimosaceae	*Prosopis chinensis* (Molina) Stuntz	Stem extract	5000 ppm	G	Adults	Mortality between 61 and 80%	-	[71]
Mimosaceae	*Prosopis chinensis* (Molina) Stuntz	Stem extract	2500 ppm	G	Adults	Mortality between 40 and 60%	-	[71]
Mimosaceae	*Prosopis chinensis* (Molina) Stuntz	Fruit extract	5000 ppm	G	Adults	More than 80% of mortality	-	[71]
Mimosaceae	*Prosopis chinensis* (Molina) Stuntz	Fruit extract	2500 ppm	G	Adults	Mortality between 61 and 80%	-	[71]
Myrtaceae	*Callistemon viminals* (Sol. ex Gaertn.) G. Don	Leaf extract	40.66 µL/L air	A	Adult females	LD_50_	1,8-cineole (71.77%)	[55]
Myrtaceae	*Eucalyptus camaldulensis* Dehnh.	Leaf extract	18–50%	A,B	Adult females	Mortalities of 62.61% and 55.57%, respectively	-	[60]
Myrtaceae	*Eucalyptus camaldulensis* Dehnh.	Flower extract	20–50%	A,B	Adult females	Mortalities of 51.91% and 47.15%, respectively	-	[60]
Myrtaceae	*Eucalyptus citriodora* Hook	Essential oil	19 × 10^−3^ µL/mL of air	J	Adults	100% of mortality	-	[42]
Myrtaceae	*Eucalyptus citriodora* Hook	Essential oil	19.3 µg/cm^3^		Adults	LD_50_	-	[68]
Myrtaceae	*Eucalyptus ghomphocephala* A. Cunn. Ex. DC.	Essential oil	-	D	Larvae and adults	Mortalities of 60% (larvae) and 34% (adults)	-	[88]
Myrtaceae	*Eucalyptus globulus* Labill.	Essential oil	19 × 10^−3^ µL/mL of air	J	Adults	89% of mortality	-	[42]
Myrtaceae	*Eucalyptus globulus* Labill.	Essential oil	0.1%	G	Adult females	19.7% of mortality	-	[112]
Myrtaceae	*Eucalyptus microtheca* F. Muell.	Essential oil vapors from fruits and leaves	1.52 µL/L (females), 5.7 µL/L (eggs)	J	Eggs and adults	LD_50_	-	[84]
Myrtaceae	*Eucalyptus microtheca* F. Muell.	Fruits and leaves essential oils	0.56% (leaves), 2.36% (fruits)	E	Adults	LD_50_	-	[83]
Myrtaceae	*Eucalyptus oleosa* L.	Essential oil	2.42 µL/L air	J	Adult females	LD_50_	1,8-Cineole (31.96%), α-pinene (15.25%), trans-anethole (7.32%)	[132]
Myrtaceae	*Eucalyptus radiata* Sieber ex. DC.	Essential oil	0.1%	G	Adult females	27.9% of mortality	-	[112]
Myrtaceae	*Eucalyptus torquata* L.	Essential oil	3.59 µL/L air	J	Adult females	LD_50_	1,8-cineole (28.57%), α-pinene (15.74%), globulol (13.11%)	[132]
Myrtaceae	*Eucapyptus* sp.	Essential oil	2.18–7.33%	C	Adults and eggs	LD_50_	-	[89]
Myrtaceae	*Eugenia caryophyllata* Thunb.	Bud and leaf essential oils	19 × 10^−3^ µL/mL of air	J	Adults	Mortalities of 80% (buds) and 66% (leaves)	-	[42]
Myrtaceae	*Eugenia caryophyllata* Thunb.	Essential oil	23.6 µg/cm^3^	C,J	Adults	LD_50_	-	[68]
Myrtaceae	*Melaleuca alternifolia* Maiden & Betche ex. Cheel	Essential oil	0.1%	G	Adult females	28.6% of mortality	-	[112]
Myrtaceae	*Melaleuca leucadendron* L.	Essential oil	0.1%	G	Adult females	23.5% of mortality	-	[112]
Myrtaceae	*Melaleuca viridiflora* Sol. Ex. Gaertn.	Essential oil	0.1%	G	Adult females	26.8% of mortality	-	[112]
Myrtaceae	*Myrtus communis* L.	Essential oil	-	D	Larvae and adults	Mortalities of 82% (larvae) and 47% (adults)	-	[88]
Myrtaceae	*Pimenta racemosa* (Mill.) J.W. Moore	Essential oil	19 × 10^−3^ µL/mL of air	J	Adults	60% of mortality	-	[42]
Myrtaceae	*Syzygium aromaticum* (L.) Merr. & L.M. Perry	Essential oil from flower buds	6.13 µL/L air	C,J	Adult females	LD_50_	eugenol (78.5%), β-caryophyllene (13.8%)	[85]
Myrtaceae	*Syzygium aromaticum* (L.) Merr. & L.M. Perry	Essential oil	0.1%	G	Adult females	41.3% of mortality	-	[112]
Myrtaceae	*Syzygium cumini* (L.) Skeels	Ethanolic, hexane and ether ethyl acetate extracts	75. 150 and 300 µg/mL	C	Adult females	Mortalities of 98.5% (ethanolic extract), 94% (hexane extract) and 90% (ether-ethyl acetate extract)	-	[108]
Nitrariaceae	*Peganum harmala* L.	Essential oil	-	D	Larvae and adults	Mortalities of 34% (larvae) and 12% (adults)	-	[88]
Nitrariaceae	*Peganum harmala* L.	Aerial part extract	1%	G	Adult females	Mortality between 20 and 50%	-	[99]
Nyctaginaceae	*Bougainvilleae spectabilis* Willd.	Leaf extract	5000 ppm	G	Adults	Mortality between 61 and 80%	-	[71]
Nyctaginaceae	*Bougainvilleae spectabilis* Willd.	Leaf extract	2500 ppm	G	Adults	Mortality between 61 and 80%	-	[71]
Papaveraceae	*Chelidonium majus* L.	Aerial part extract	1%	G	Adult females	Mortality between 0 and 20%	-	[99]
Papaveraceae	*Papaver pavoninum* Schrenk.	Aerial part extract	1%	G	Adult females	Mortality between 0 and 20%	-	[99]
Papaveraceae	*Papaver rhoeas* L.	Essential oil	-	D	Larvae and adults	Mortalities of 43% (larvae) and 34% (adults)	-	[88]
Papaveraceae	*Papaver rhoeas* L.	Aerial part extract	1%	G	Adult females	Mortality between 80 and 100%	-	[99]
Papaveraceae	*Roemeria refracta* DC.	Aerial part extract	1%	G	Adult females	Mortality between 50 and 80%	-	[99]
Pinaceae	*Cedrus atlantica* (Endl.) Manetti ex. Carriére	Essential oil	0.1%	G	Adult females	12.4% of mortality	-	[112]
Pinaceae	*Picea schrenkiana* Fisch & Mey.	Leaf extract	1%	G	Adult females	Mortality between 50 and 80%	-	[99]
Pinaceae	*Pinus sylvestris* L.	Essential oil	0.1%	G	Adult females	50.4% of mortality	-	[112]
Piperaceae	*Piper aduncum* L.	Leaf essential oil compounds	-	J	-	Mortality effect [(E)-nerolidol, α–Humulene and β-caryophyllene)] and repellence (β-caryophyllene)	(*E*)-nerolidol, α–Humulene and β-caryophyllene	[109]
Piperaceae	*Piper nigrum* L.	Essential oil	0.1%	G	Adult females	22.8% of mortality	-	[112]
Plantaginaceae	*Globularia alypum* L.	Essential oil	-	D	Larvae and adults	Mortalities of 8% (larvae) and 2% (adults)	-	[88]
Plantaginaceae	*Plantago major* L.	Aerial part extract	1%	G	Adult females	Mortality between 80 nd 100%	-	[99]
Plumbaginaceae	*Limonium tianschanicum* Lincz.	Aerial part extract	1%	G	Adult females	Mortality between 0 and 20%	-	[99]
Polygonaceae	*Polygonum aviculare* L.	Aerial part extract	1%	G	Adult females	Mortality between 20 and 50%	-	[99]
Polygonaceae	*Polygonum toktoquilicum* Lazkov	Root extract	1%	G	Adult females	Mortality between 0 and 20%	-	[99]
Polygonaceae	*Rumex acetosa* L.	Aerial part extract	1%	G	Adult females	Mortality between 20 and 50%	-	[99]
Primulaceae	*Anagallis arvensis* L.	Aerial part extract	1%	G	Adult females	Mortality between 50 and 80%	-	[99]
Ranunculaceae	*Aconitum soongaricum* Stapf	Aerial part extract	1%	G	Adult females	Mortality between 50 and 80%	-	[99]
Ranunculaceae	*Adonis parviflora* Fisch.	Aerial part extract	1%	G	Adult females	Mortality between 0 and 20%	-	[99]
Ranunculaceae	*Ceratocephallus testiculata* (Crantz.) Bess.	Aerial part extract	1%	G	Adult females	Mortality between 0 and 20%	-	[99]
Ranunculaceae	*Clematis orientalis* L.	Seed extract	1%	G	Adult females	Mortality between 50 and 80%	-	[99]
Ranunculaceae	*Clematis songarica* Bge.	Seed extract	1%	G	Adult females	Mortality between 20 and 50%	-	[99]
Ranunculaceae	*Nigella sativum* L.	Seed extract	708.57 ppm	G	Adult females	LD_50_	-	[91]
Ranunculaceae	*Ranunculus polyanthemus* L.	Aerial part extract	1%	G	Adult females	Mortality between 0 and 20%	-	[99]
Rosaceae	*Geum urbanum* L.	Aerial part extract	1%	G	Adult females	Mortality between 0 and 20%	-	[99]
Rosaceae	*Padus avium* Mill.	Leaf extract	1%	G	Adult females	Mortality between 0 and 20%	-	[99]
Rosaceae	*Prunus laurocerasus* L.	Leaves, flower and seed extract	12% v/w	A,C	Eggs and adult females	Mortality between 37 and 100%	-	[54]
Rubiaceae	*Boirerio radiata*	Whole plant extract	5000 ppm	G	Adults	Mortality between 40 and 60%	-	[71]
Rubiaceae	*Boirerio radiata*	Whole plant extract	2500 ppm	G	Adults	Mortality between 40 and 60%	-	[71]
Rubiaceae	*Galium verum* L.	Aerial part extract	1%	G	Adult females	Mortality between 50 and 80%	-	[99]
Rubiaceae	*Gardenia jasminoides* J. Ellis	Fruits extract	10000 ppm	I,J	Adult females and nymphs	49 and 66% of mortality, respectively	-	[133]
Rutaceae	*Citrus aurantium* L.	Essential oil	19 × 10^−3^ µL/mL of air	J	Adults	68% of mortality	-	[42]
Rutaceae	*Citrus aurantium* L.	Essential oil	-	D	Larvae and adults	Mortalities of 63% (larvae) and 55% (adults)	-	[88]
Rutaceae	*Citrus aurantium* L.	Fruit epicarp essential oil	1%	I,J	Adult females	Repellent effect due to all 27 identified compounds	*d*-limonene	[67]
Rutaceae	*Citrus aurantium* L. *var. Armara*	Essential oil	0.1%	G	Adult females	21.4% of mortality	-	[112]
Rutaceae	*Citrus bergamia* Risso & Poit.	Essential oil	19 × 10^−3^ µL/mL of air	J	Adults	87% of mortality	-	[42]
Rutaceae	*Citrus bergamia* Risso & Poit.	Essential oil	0.1%	G	Adult females	11% of mortality	-	[112]
Rutaceae	*Citrus limon* (L.) Burm. F.	Essential oil	0.1%	G	Adult females	34.9% of mortality	-	[112]
Rutaceae	*Citrus paradisi* Macfad	Essential oil	6.96 µL/L air	A	Adult females	LD_50_	limonene (74.29%)	[55]
Rutaceae	*Citrus paradisi* Macfad.	Essential oil	0.1%	G	Adult females	30.6% of mortality	-	[112]
Rutaceae	*Citrus sinensis* Osbeck	Essential oil	19 × 10^−3^ µL/mL of air	J	Adults	61% of mortality	-	[42]
Rutaceae	*Citrus sinensis* Osbeck	Fruit epicarp essential oil	1%	I,J	Adult females	Repellent effect due to all 27 identified compounds	*d*-limonene	[67]
Rutaceae	*Citrus sinensis* Osbeck	Essential oil	0.1%	G	Adult females	45.6% of mortality	-	[112]
Rutaceae	*Haplophyllum tuberculatum* (Forssk.) A. Juss.	Essential oil	-	D	Larvae and adults	Mortalities of 94% (larvae) and 93% (adults)	-	[88]
Rutaceae	*Ruta chalepensis* L.	Essential oil	-	D	Larvae and adults	Mortalities of 66% (larvae) and 61% (adults)	-	[88]
Rutaceae	*Zanthoxylum armatum* DC.	Leaf extract	5000 and 10000 ppm	G	Adults	Mortalities of 36% and 39%, respectively	-	[111]
Santalaceae	*Santalum* sp.	Essential oil	0.1%	G	Adult females	87.2% of mortality and fecundity decrease	-	[112]
Scrophulariaceae	*Calceolaria andina* Benth	Two extract compounds	80 and 30 ppm	G	-	LD_50_	2-(1,1-dimethylprop-2-enyl)-3-hydroxi-1,4-naphthoquinone and 2-acetoxy-3-(1,1-dimethylprop-2-enyl)-1,4-naphthoquinone	[113]
Scrophulariaceae	*Verbascum thapsus* L.	Aerial part extract	1%	G	Adult females	Mortality between 20 and 50%	-	[99]
Simarubaceae	*Ailanthus altissima* (Mill.) Swingle	Leaf extract	1%	G	Adult females	Mortality between 80 and 100%	-	[99]
Simarubaceae	*Quassia* sp.	Aerial part extract	10000 ppm and 47 ppm (chaparinone)	C	-	LD_50_ (chaparinone)	Chaparinone quasinoid	[114]
Solanaceae	*Capsicum annuum* L.	Aerial part extract	1%	G	Adult females	Mortality between 80 and 100%	-	[99]
Solanaceae	*Capsicum annuum* L.	Fruit extract	-	B,J	Adult females	45% of mortality	-	[116]
Solanaceae	*Capsicum baccatum* L.	Fruit extract	-	B,J	Adult females	Repellent effect	-	[116]
Solanaceae	*Capsicum chinense* Jacq.	Fruit extract	-	B,J	Adult females	Repellent effect	-	[116]
Solanaceae	*Capsicum frutescens* L.	Fruit extract	-	B,J	Adult females	Repellent effect	-	[116]
Solanaceae	*Datura stramonium* L.	Seed and leaf extracts	167.25 (leaves) and 145.75 (seeds) mg/L	C,D	Adults	Mortalities of 98% (leaves) and 25% (seeds)	-	[5]
Solanaceae	*Hyoscyamus niger* L.	Aerial part extract	1%	G	Adult females	Mortality between 50 and 80%.	-	[99]
Solanaceae	*Lycopersicon hirsutum* Dunal	-	-	J	Adult females	Repellent activity	Dihidrofarnesoic acid	[115]
Solanaceae	*Solanum nigrum* L.	Flower and leaf extracts	12–50%	A,B	Adult females	Mortalities of 69.88% and 79.36%, respectively	-	[60]
Solanaceae	*Solanum nigrum* L.	Fruit extract	19–50%	A,B	Adult females	Mortalities of 68.78% and 53.29%, respectively	-	[60]
Solanaceae	*Solanum nigrum* L.	Leaf extract	1%	G	Adult females	Mortality between 20 and 50%	-	[99]
Solanaceae	*Solanum nigrum* L.	Leaf extract	279.69 µg/mL	C,G	Adult females	LD_50_ after 72 h	-	[134]
Sterculiaceae	*Waltheria indica* L.	Whole plant extract	5000 ppm	G	Adults	Mortality between 61 and 80%	-	[71]
Sterculiaceae	*Waltheria indica* L.	Whole plant extract	2500 ppm	G	Adults	Mortality between 40 and 60%	-	[71]
Styracaceae	*Styrax officinalis* L.	Seed cover extract	16–50%	A,B	Adult females	Mortalities of 64.11% and 73.25%, respectively	-	[60]
Styracaceae	*Styrax officinalis* L.	Seed extract	21–50%	A,B	Adult females	Mortalities of 68.17% and 31.28%, respectively	-	[60]
Umbelliferae	*Angelica tschimganica* (Korov.) B. Tikhom.	Aerial part extract	1%	G	Adult females	Mortality between 20 and 50%	-	[99]
Umbelliferae	*Conium maculatum* L.	Seed extract	1%	G	Adult females	Mortality between 20 and 50%	-	[99]
Umbelliferae	*Dorema microcarpum* Korov.	Aerial part extract	1%	G	Adult females	Mortality between 20 and 50%	-	[99]
Umbelliferae	*Ferula foetida* (Bunge) Regel.	Root extract	1%	G	Adult females	Mortality between 0 and 20%	-	[99]
Umbelliferae	*Ferula foetidissima* Regel. & Schmahl.	Root extract	1%	G	Adult females	Mortality between 20 and 50%	-	[99]
Umbelliferae	*Ferula inciso-serrata* M. Pimen. & Baranova	Aerial part and root extracts	1%	G	Adult females	Mortality between 0 and 20%	-	[99]
Umbelliferae	*Heracleum dissectum* Ledeb.	Aerial part extract	1%	G	Adult females	Mortality between 20 and 50%	-	[99]
Umbelliferae	*Mediasia macrophylla* (Regel. & Schmahl.) M. Pimen.	Aerial part extract	1%	G	Adult females	Mortality between 50 and 80%	-	[99]
Umbelliferae	*Prangos lipskyi* Korov	Root extract	1%	G	Adult females	Mortality between 80 and 100%	-	[99]
Urticaceae	*Urtica pilulifera* L.	Essential oil	-	D	Larvae and adults	Mortalities of 49% (larvae) and 46% (adults)	-	[88]
Valerianaceae	*Valeriana officinalis* L.	Aerial part extract	1%	G	Adult females	Mortality between 20 and 50%.	-	[99]
Verbenaceae	*Lantana camara* L.	Essential oil	-	D	Larvae and adults	Mortalities of 2% (larvae) and 1% (adults)	-	[88]
Verbenaceae	*Lippia origanoides* H.B.K.	Essential oil	25.1 μg/mL	J	Adult females	LD_50_	carvacrol (48.31%), *p*-cymene (9.11%), thymol (8.78%)	[135]
Verbenaceae	*Lippia sidoides* Cham.	Essential oil	0.01 µL/L (extract). 0.001 µL/L (thymol). 3.02 µL/L (p-cymene). 0.08 µL/L (β-caryophyllene) and 0.036 µL/L (carvacrol)	J	Adult females	LD_50_	Thymol, *p*-cymene, β-caryophyllene and carvacrol	[117]
Zingiberaceae	*Elettaria cardamomum* (L.) Maton	Essential oil	19 × 10^−3^ µL/mL of air	J	Adults	87% of mortality	-	[42]
Zingiberaceae	*Zingiber officinale* Rosc.	Essential oil	0.1%	G	Adult females	11.9% of mortality	-	[112]
Zygophyllaceae	*Balanites aegyptiaca* (L.) Delile	Leaf extract	5000 ppm	G	Adults	More than 80% of mortality	-	[71]
Zygophyllaceae	*Balanites aegyptiaca* (L.) Delile	Leaf extract	2500 ppm	G	Adults	Mortality between 61 and 80%	-	[71]

^a^ This column includes the bioassays used in each study, which are coded as follows: A = slide dip, B = petri dish, C = leaf disc direct (LDD); D = leaf disc residue (LDR); E = leaf disc direct potter tower (LDD-PT); F = leaf disc residue potter tower (DR-PTM); G = leaf disc residue dipping (LDR-D); H = leaf absorption test; I = whole plant direct; J = filter paper difussion (fumigant).
